# Quantifying microbial robustness in dynamic environments using microfluidic single-cell cultivation

**DOI:** 10.1186/s12934-024-02318-z

**Published:** 2024-02-09

**Authors:** Luisa Blöbaum, Luca Torello Pianale, Lisbeth Olsson, Alexander Grünberger

**Affiliations:** 1 Multiscale Bioengineering, Technical Faculty, Bielefeld University, Bielefeld, Germany; 2 CeBiTec, Bielefeld University, Bielefeld, Germany; 3 Industrial Biotechnology Division, Department of Life Sciences, Chalmers University of Technology, Gothenburg, Sweden; 4 Microsystems in Bioprocess Engineering, Institute of Process Engineering in Life Sciences, Karlsruhe Institute of Technology, Karlsruhe, Germany

**Keywords:** *Saccharomyces cerevisiae*, Population heterogeneity, Dynamic environments, Scale-down, Biosensors, Live-cell imaging, Microfluidic single-cell cultivation, Nutrient oscillation

## Abstract

**Background:**

Microorganisms must respond to changes in their environment. Analysing the robustness of functions (i.e. performance stability) to such dynamic perturbations is of great interest in both laboratory and industrial settings. Recently, a quantification method capable of assessing the robustness of various functions, such as specific growth rate or product yield, across different conditions, time frames, and populations has been developed for microorganisms grown in a 96-well plate. In micro-titer-plates, environmental change is slow and undefined. Dynamic microfluidic single-cell cultivation (dMSCC) enables the precise maintenance and manipulation of microenvironments, while tracking single cells over time using live-cell imaging. Here, we combined dMSCC and a robustness quantification method to a pipeline for assessing performance stability to changes occurring within seconds or minutes.

**Results:**

*Saccharomyces cerevisiae* CEN.PK113-7D, harbouring a biosensor for intracellular ATP levels, was exposed to glucose feast-starvation cycles, with each condition lasting from 1.5 to 48 min over a 20 h period. A semi-automated image and data analysis pipeline was developed and applied to assess the performance and robustness of various functions at population, subpopulation, and single-cell resolution. We observed a decrease in specific growth rate but an increase in intracellular ATP levels with longer oscillation intervals. Cells subjected to 48 min oscillations exhibited the highest average ATP content, but the lowest stability over time and the highest heterogeneity within the population.

**Conclusion:**

The proposed pipeline enabled the investigation of function stability in dynamic environments, both over time and within populations. The strategy allows for parallelisation and automation, and is easily adaptable to new organisms, biosensors, cultivation conditions, and oscillation frequencies. Insights on the microbial response to changing environments will guide strain development and bioprocess optimisation.

**Graphical Abstract:**

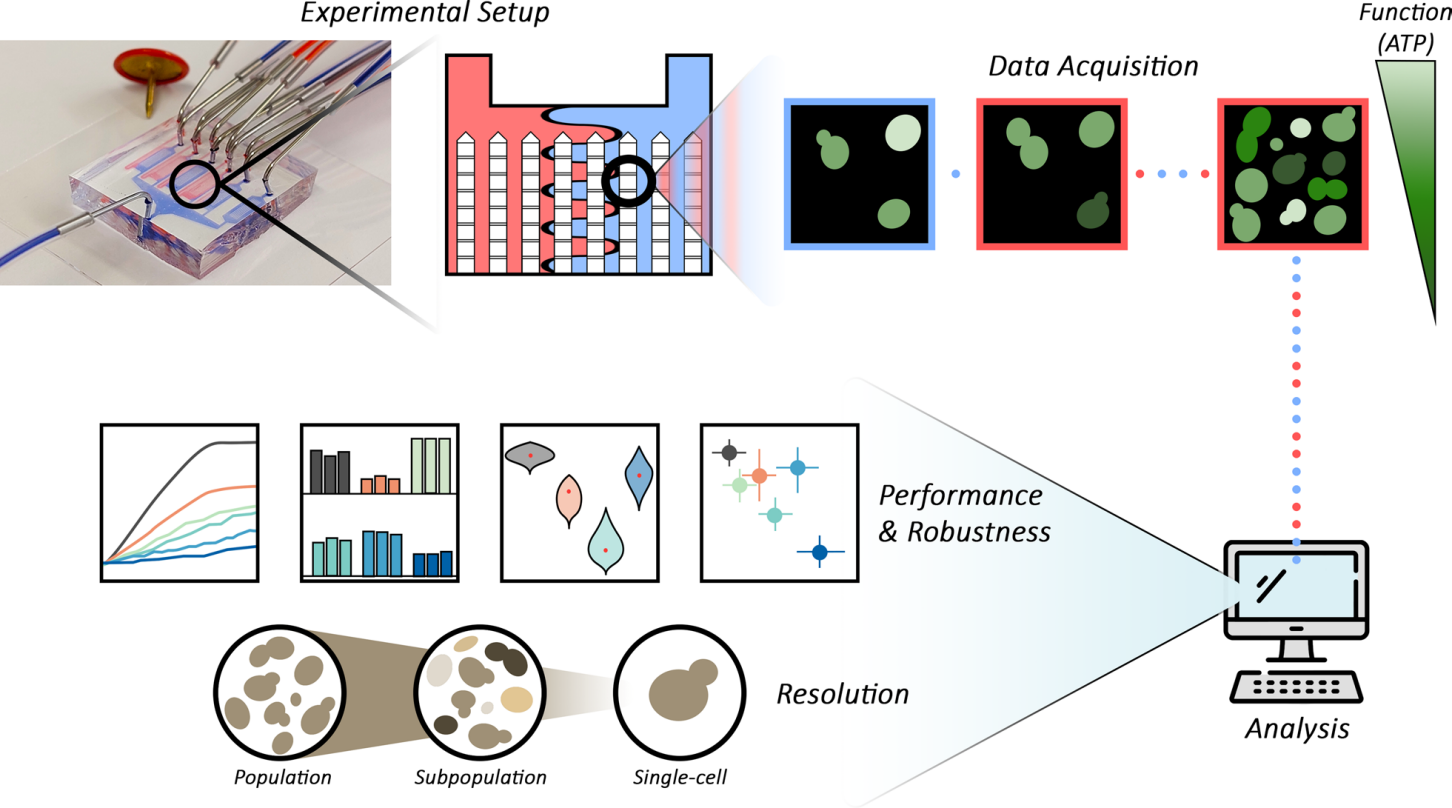

**Supplementary Information:**

The online version contains supplementary material available at 10.1186/s12934-024-02318-z.

## Background

Microorganisms encounter dynamic and heterogeneous environments, both in their natural habitat and in laboratory or industrial settings, which they experience as environmental change within a few seconds or even days [1, 2]. In large-scale industrial bioreactors, insufficient mixing triggers gradients in multiple process parameters [2], which can decrease productivity [3], increase both cell viability [4] and metabolic cost [5], and favour population heterogeneity [6]. This, in turn, results in poorly reproducible or predictable behaviour during scale-up, likely diminishing profitability [7]. Reproducibility and transferability of a bioprocess can be increased by selecting or engineering microorganisms with stable production under large-scale perturbations [8]. Robustness refers to the stability of a function (e.g. yield, titre or rates) in a system (e.g. a microorganism) subjected to perturbations [8]. Comparison of robustness for specific functions across microorganisms is limited by the dearth of quantification methods. A recent study reviewed mathematical approaches for robustness quantification and derived a formula from the Fano factor, the variance-to-mean ratio, to compare the robustness of process-relevant functions for different yeast strains within a set of perturbations [9]. This equation helped identify trade-offs between robustness and performance [10], thereby providing information on function stability over time or across populations [11].

Performance in dynamic environments with large time constants (e.g. days or longer) has received more attention than that regulated by small time constants (e.g. seconds or minutes). An example of the latter is represented by substrate, pH or gas gradients that form in a large reactor [12]. Instead, examples of the former include decreasing substrate concentration and increasing product concentration during cultivation, which cause product inhibition [13] or detoxification of inhibitory compounds in the substrate [14]. The effect of large time constant dynamics on microorganisms can be assessed using shaking flasks or microtiter plates, in which the culture broth is perfectly mixed [9]; whereas those involving small time constants can be established in scale-down bioreactors. The latter simulate large-scale gradients by circulating the microbial culture between multiple compartments with different cultivation conditions or by altering conditions in a continuous bioreactor [15]. The second approach was used to investigate the effect of changes between feast, nutrient limitation, and starvation conditions on the metabolism and gene expression of *Saccharomyces cerevisiae* strains CEN.PK113-7D [5, 16] and Ethanol Red [17]. Scale-down approaches are a valuable tool to test and identify targets for improving microbial production performance against dynamic perturbations. In *Escherichia coli*, the deletion of genes induced repeatedly during glucose oscillations decreased maintenance costs while improving stability of the production phenotype [18]. However, scale-down bioreactors have some limitations. The environmental dynamics in them are often dictated by cellular metabolism, with the environment changing in response to consumption and production of substrates and byproducts. Furthermore, output data are mostly population-averaged, hiding effects of population heterogeneity on bioprocess performance [19]. The metabolic burden of production, limiting growth conditions and numerous generation during the seed train to industrial size fermentations drive the selection process toward subpopulations with a higher fitness in these environments [20]. As a result, a decline in productivity and product yield can often be observed [21].

Although single-cell resolution can be achieved through automated real-time flow cytometry and population heterogeneity within each sample can be determined [22], tracking individual cells in time is not possible. Moreover, running scale-down reactors in parallel with different conditions or strains is challenging due to space and resource requirements. Single-cell resolution and application of metabolism-independent varying environments can be achieved with dynamic microfluidic single-cell cultivation (dMSCC) [23]. In perfusion-based microfluidic systems, a maximum of 150–1000 microbial cells can be cultivated and trapped in one monolayer-growth chambers with femto- to nanolitre volumes, thereby achieving excellent heat and mass transfer that create well-defined environments [24]. Valves or on-chip laminar flow-control [25] can alter cultivation conditions within a few seconds, independently from microbial consumption or production rates [23, 26]. As these changes are within the time frame of large-scale reactor dynamics [27], dMSCC can be used to simulate such settings, although limitations in stress amplitude modulation, dynamics of dissolved gases, and multi-parameter dynamics remain [28]. While the use of dMSCC to mimic large-scale gradients is fairly new, it has been applied successfully to investigate glycolytic oscillation [29], growth synchronisation [30], and ageing [31] in *S. cerevisiae*.

In the present study, we aimed to develop a method to investigate performance and robustness of desired functions in a wide range of rapid environmental changes. To this end, we combined the principle of dynamic environments of dMSCC [23] with a previously-published robustness quantification method [9], and grew the laboratory *S. cerevisiae* CEN.PK113-7D strain under feast-starvation oscillations with regular changes every 1.5–48 min. Glucose gradients are a common perturbation in industrial bioprocesses [2, 32]. Multiple cellular functions were monitored either via phase-contrast microscopy (growth, cell area, and circularity) or via the ratiometric fluorescent biosensor QUEEN-2m, which monitors intracellular ATP [33, 34]. Using tailored semi-automated image and data analyses in Fiji [35] and R [36], respectively, we achieved a level of resolution spanning the population, subpopulation, and single-cell levels. Distinct feast-starvation-cycles caused different physiological responses including frequency-dependant reduction of the specific growth rate, as well as morphological changes. Moreover, physiological characterisation and performance analysis were coupled with robustness quantification to investigate the stability of functions over time and within populations, thereby assessing population heterogeneity. The presented pipeline offers a powerful approach to study dynamic environments and a starting point for industrial strain selection already at the laboratory scale.

## Methods

### Strain, media composition, and cultivation

The yeast strain CEN.PK113-7D (MATa URA3 HIS3 LEU2 TRP1 MAL2-8c SUC2) [37] bearing the ATP-biosensor QUEEN-2m [33, 34] was cultivated in synthetic defined minimal Verduyn (“Delft”) medium [38] with pH adjusted to 5 with KOH. The medium contained 20 g/L glucose, 5 g/L (NH_4_)_2_SO_4_, 3 g/L KH_2_PO_4_, 1 g/L MgSO_4_ · 7H_2_O, 1 mL/L trace metal solution, and 1 mL/L vitamin solution [34]. For the preculture cultivated in shaking flasks, 5.9 g/L succinic acid was added as buffer [39]. Starting from a cryostock, 10 mL of preculture was inoculated in a 100 mL-baffled shaking flask and cultivated for 16 h at 30 °C, 120 rpm, and a shaking throw of 25 mm. To simulate starvation, glucose was substituted by water.

### Dynamic microfluidic single-cell cultivation

**Fig. 1:**
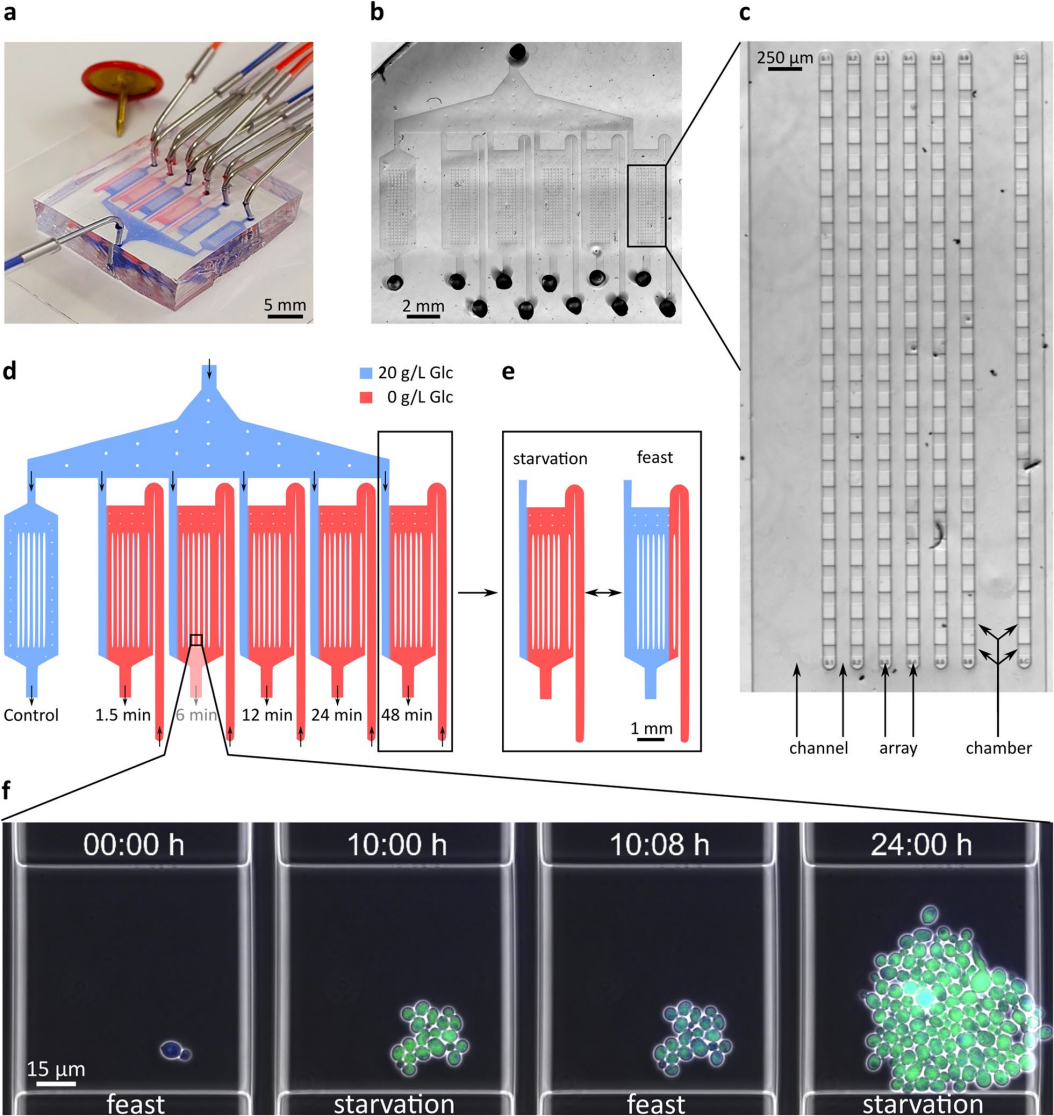
Dynamic microfluidic single-cell cultivation. Photos of (**a**) the PDMS-based dynamic microfluidic cultivation chip, (**b**) the microfluidic structure and (**c**) a single oscillation-structure. Each structure contains six arrays of 23 cultivation chambers in the oscillation region and one separate array to the right that acted as control for the starvation condition. (**d**) Feast medium, containing glucose (blue), and starvation medium (red) are introduced via inlets. The left cultivation structure is constantly perfused with feast medium. (**e**) The flow profile in the oscillation structures can be switched between feast and starvation. (**f**) In each chamber, yeast cells grew in a monolayer under constant perfusion with either feast or starvation medium that was switched every 1.5 min

The microfluidic structure (Fig. 1a, b) consists of six connected cultivation structures, that include one dedicated positive control and five oscillation-structures. Within each structure, six arrays of 23 monolayer-growth chambers were exposed to the dynamic condition, with one additional array acting as negative control (Fig. 1c). Each monolayer-growth chamber has dimensions of 4 × 90 × 80 µm (height × width × length), while the channels have a height of 14 µm.

For the chip fabrication, a polydimethylsiloxane (PDMS) mould of the microfluidic structures was cut from a master wafer. To enable flow through the structure, inlets and outlets were punched using a biopsy puncher. The PDMS-mould and a glass slide were cleaned, and their surfaces activated using oxygen plasma. In the final assembly step, the activated PDMS was bonded to the glass slide (Fig. 1a). The reader is referred to a previous protocol for a detailed description of the fabrication and application of dynamic microfluidic cultivation systems [40].

The chip was placed in an inverted automated microscope (Nikon Eclipse Ti2, Nikon) for live-cell imaging and inoculated with yeast cells at OD_600_ ~ 0.3. The cultivation temperature inside the microscope’s incubation cage (OKO-H201, OKO Lab) was set to 30 °C. Using a 100 × oil objective (CFI P-Apo DM Lambda, Nikon), phase-contrast and fluorescent images were taken every 8 min using two filters: GFP (Ex 472/30; DM 495; Em 520/35, Nikon) and uvGFP (Ex 390/40; DM 425; Em 520/35, AHF). Phase-contrast images were captured for 100 ms at 25% intensity using the microscope’s DIA illumination, GFP images were captured for 400 ms at 25% intensity, and uvGFP images for 800 ms at 25% intensity using a LED-based light source for episcopic fluorescence (Sola SE II Set, Lumencor).

A dynamic flow profile (Fig. 1d, e) was applied using pressure-driven pumps (Line-up EZ series, Fluigent) that maintained glucose-containing medium (blue) at 100 mbar, but switched between 70 and 220 mbar to control flow of glucose-free medium. Medium was switched every 1.5, 6, 12, 24, and 48 min to cover different biological timescales [1, 41]. The above frequencies were multiples of each other, owing to the pump programming. Perturbations were initiated 4 h after the onset of cultivation. Image acquisition was terminated after 24 h and each position was saved as an individual nd2 file for automated image analysis.

### Image analysis

Image handling and analysis were performed in Fiji [35] using a semi-automated macro for an easier pipeline (https://github.com/lucatorep/Robustness_Microfluidics). All single-point nd2 files were sequentially loaded, stabilised, tilted, and cut to the size of the monolayer-growth chamber to minimise computation times. Rolling ball background subtraction was applied to the fluorescent channels to quantify the corresponding signal [33]. The resulting hyperstacks were saved as TIFF files.

We applied a StarDist 2D model for yeast cell segmentation [26]. A machine learning model was trained on our own data using the StarDist 2D ZeroCostDL4Mic notebook [42] and an augmentor algorithm [43]. The trained model “Yeast_Segmentation_v2.2” is available via GitHub (https://github.com/lucatorep/Robustness_Microfluidics).

The TrackMate Fiji plug-in was used for cell tracking and lineage reconstruction (i.e. mother-daughter relationships) [44]. The StarDist model was applied for detection with a score threshold of 0.3; while the overlap tracker served for tracking, with the settings “Precise”, “Min IoU = 0”, and “Scale factor = 1.2”. After TrackMate analysis and manual track corrections, the regions of interest and edges (representing the lineage) were exported as.txt and.csv files, respectively. The TrackMate file (.xml) was saved to enable later editing, if necessary.

### Robustness quantification

Robustness was determined as described previously [9, 11], based on Eq. 1 [9].

1 $${\varvec{R}}=\boldsymbol{ }-\frac{{{\varvec{\sigma}}}^{2}}{\overline{{\varvec{x}}} }\boldsymbol{*}\frac{1}{{\varvec{m}}}$$

Although this equation was originally used to compute the robustness of functions across different conditions, here, its versatility allowed to estimate function stability across populations and time. For robustness across populations, R(p), “σ” and “x” refer to the standard deviation and mean, respectively, of a function (ATP levels, area or circularity) across all cells at each time point. Instead, “m” refers to the mean of a function across all time points and conditions. Therefore, R(p) describes how homogeneous a function is across a cell population.

Robustness over time, R(t), identifies how stable a function is over time in each condition investigated and was computed at the population and single-cell levels. At the population level, the mean of a function across all cells at each time point was first computed; then, Eq. 1 was used to quantify R(t), with “σ” and “x” referring to the standard deviation and mean, respectively, of the population-averaged function (ATP levels, budding ratio, area or circularity) across all time points for each condition and chamber. In this case, “m” refers to the mean of a function across all time points and conditions. For R(t) at the single-cell level, “σ” and “x” refer to the standard deviation and mean, respectively, of a function (ATP levels, specific growth rate, area or circularity) across all time points for each individual cell; whereas “m” refers to the mean of a function across all cells in all conditions.

### Data and statistical analysis

Analysis of performance and robustness was carried out in R [36]. To assess growth performance, the specific growth rate µ and budding ratio were used at single-cell and population level, respectively. The specific growth rates of single cells were computed according to Eq. 2:

2 $${\varvec{\mu}}=\boldsymbol{ }\frac{\mathbf{ln}2}{{\varvec{t}}}$$

where “t” represents the time (h) between two budding events detected by TrackMate for each cell. Accordingly, if a cell budded three times, it displayed three individual specific growth rates. Artefacts in µ, such as a cell budding 30 min after inoculation of the chamber (giving a µ of 1.3 h^−1^), were removed by setting a threshold of 0.6 h^−1^ (all values above were eliminated) based on maximum specific growth rate of yeast being around 0.5 h^−1^.

The budding ratio represents the number of buds per cell at any given time point and was computed according to Eq. 3:

3 $${\varvec{B}}{\varvec{u}}{\varvec{d}}{\varvec{d}}{\varvec{i}}{\varvec{n}}{\varvec{g}}\,\boldsymbol{ }{\varvec{R}}{\varvec{a}}{\varvec{t}}{\varvec{i}}{\varvec{o}}=\boldsymbol{ }\frac{{({\varvec{n}}{\varvec{o}}.\boldsymbol{ }{\varvec{b}}{\varvec{u}}{\varvec{d}}{\varvec{s}})}_{{\varvec{t}}({\varvec{n}})}}{{({\varvec{n}}{\varvec{o}}.\boldsymbol{ }{\varvec{c}}{\varvec{e}}{\varvec{l}}{\varvec{l}}{\varvec{s}})}_{{\varvec{t}}({\varvec{n}}-1)}}$$

Here, the number of new buds at a given time point “n” was divided by the number of cells at the previous time point “n-1”. The time between time points was 8 min. This approach was used for two reasons. First, chambers might fill up completely causing excess cells to be flushed away and preventing an estimation of specific growth rate using linear regression of the semi-logarithmic cell count. Instead, the budding ratio allows to estimate replication events even when the chamber is full. Second, it improves monitoring over time and at each time point of long oscillations because, unlike the specific growth rate derived from single-cell doublings, it does not average growth over time.

ATP levels were computed by dividing the uvGFP signal by the GFP signal of any given cell [33].

Error bars generally represented the standard deviation for the average robustness or performance of each replicate chamber. Whenever statistical tests were performed, they are stated in the legend of the corresponding figure. Pairwise comparisons were carried out using unpaired Student’s *t*-test (e.g. each feast-starvation oscillation with respect to the control). ANOVA was used to test differences among performance means within subpopulations (between 1 and 4) in each chamber. Statistical significance was defined as follows: *p ≤ 0.05, **p ≤ 0.01, ***p ≤ 0.001, and ****p ≤ 0.0001.

## Results

### Experimental design and development of a semi-automated pipeline

In the present study, dMSCC was employed to cultivate the laboratory *S.* *cerevisiae* CEN.PK113-7D strain under rapidly changing conditions (Additional file 1: Video S1). Further development of the dMSCC chip allowed the simultaneous assessment of five independent media oscillation frequency (Fig. 1d), rather than the previous three [23], increasing the experimental throughput significantly. To simulate a process parameter gradient relevant in large-scale reactors, we chose to oscillate glucose between 20 g/L (feast) and 0 g/L (starvation). How fast microorganisms react to environmental oscillations affects the behaviour of a specific function [1]. By switching media every 1.5, 6, 12, 24 or 48 min, several timescales were covered, ranging from transcription and mRNA degradation (seconds) to protein processing (minutes) and cell division (hours) [1, 41]. Even though oscillation frequencies differed, the total time spent under starvation or feast conditions was the same for all cells.

**Fig. 2:**
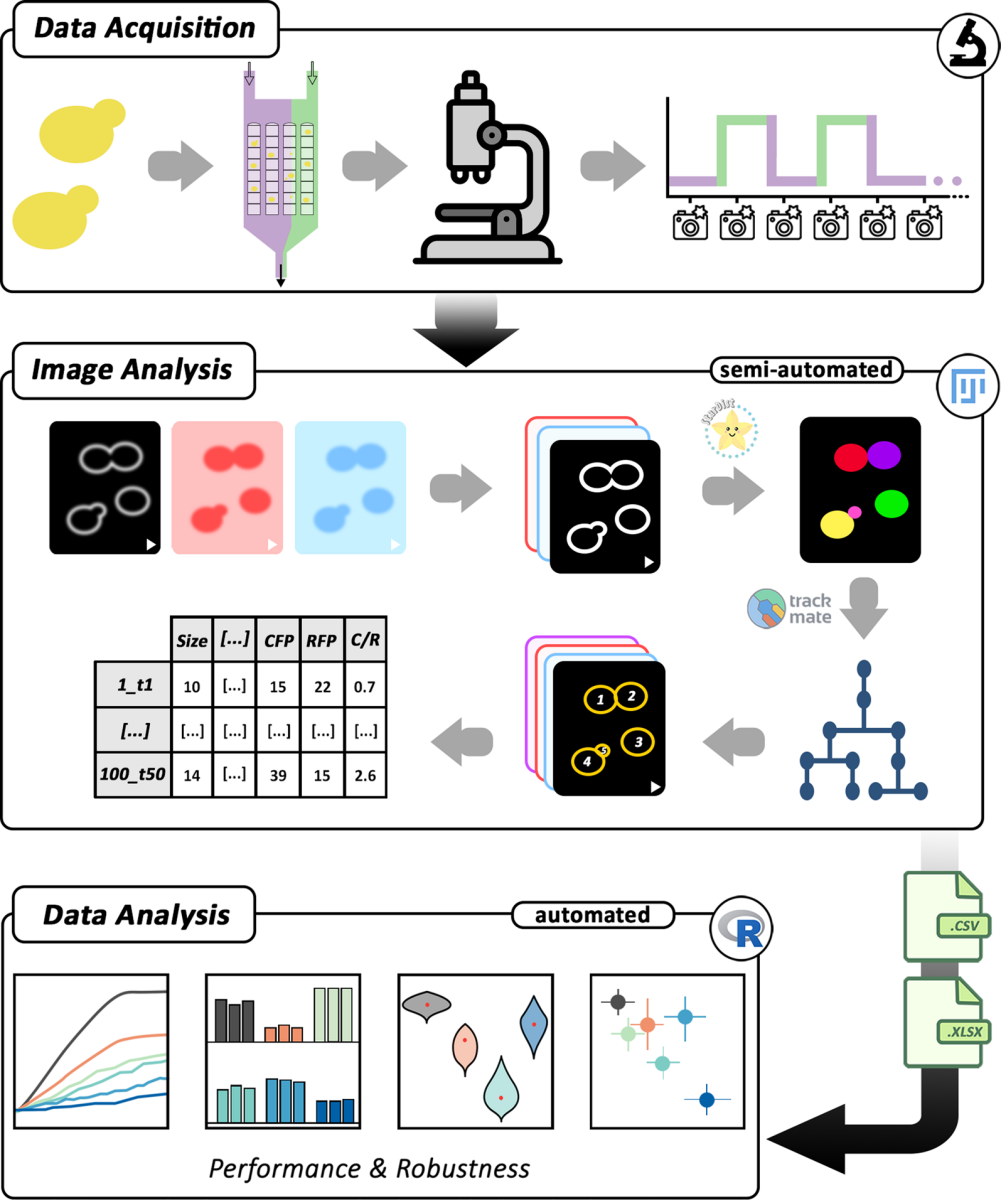
Three-step pipeline combining dMSCC and robustness analysis. The pipeline comprised three steps: data acquisition, image analysis, and data analysis. In the first step, dMSCC was performed on S. cerevisiae subjected to feast-starvation oscillations (Additional file 1: Video S1). Raw image data from live-cell imaging were pre-processed in Fiji, segmented with StarDist-2D, and single cells were tracked using TrackMate. Shape descriptors and fluorescence values were extracted for the segmented cells. Performance and robustness of the collected data were analysed in R

Physiological functions, such as specific growth rate, morphology (cell area and circularity) and ATP level (Fig. 2) were determined. Intracellular ATP was monitored via the genome-integrated fluorescent ratiometric biosensor QUEEN-2m [34]. Growth and morphology were captured by phase-contrast microscopy (Additional file 2: Figs S1, S2,). First, imaging conditions were set to capture the response within a suitable timeframe, avoiding overexposure to fluorescent light and preventing phototoxicity [45]. Preliminary experiments suggested monitoring the cellular response every 8 min, which was sufficient to capture the biosensor’s behaviour, as well as slower processes such as growth. The response time, range, and time to a new steady-state of the biosensor were tested with a temporal resolution of 3.5 s (Additional file 2: Fig S3). The sensor reacted within the next timeframe after medium switching and a new steady-state was reached within 68 s. This behaviour was reproducible over the course of 2 h. Frequencies beyond 8 min were not feasible for long-term live-cell imaging, as phototoxicity significantly influenced the specific growth rate (data not shown). Even though exposure to fluorescent light inevitably affects cell metabolism, all samples were exposed for the same time, thus enabling comparison within the study. Moreover, imaging settings for QUEEN-2m were chosen to minimise the impact on the maximal specific growth rate under control conditions [34].

To perform image analysis and handle the large sets of raw data generated, we developed a computational pipeline for both image and data analysis. This pipeline allowed to analyse and match the increased throughput possible from the new dMSCC chip layout. For image analysis, a semi-automated macro was developed in Fiji [35]. Accurate cell segmentation and single-cell tracking were crucial points in this step. The StarDist-2D plug-in [46] was used for segmentation. It employed a machine learning model that was trained on phase-contrast images of yeast cells. Canonical thresholding methods were not possible due to the presence of organelles in eukaryotic cells. Single-cell tracking and determination of mother-daughter relationships (lineage reconstruction) were achieved using TrackMate [44]. Even though the segmentation and tracking steps were automated, the user was required to give the initial input parameters for each chamber separately, thus making this step semi-automated. Manual checking and editing of tracking and lineage reconstruction were necessary to ensure data quality. In the last step, automated data analysis was carried out in R for performance and robustness quantification [9] at population, subpopulation, and single-cell level.

### Yeast physiological response to dynamic feast-starvation oscillations

The specific growth rate and budding ratio were determined by dMSCC at the single-cell and population level (see Methods), along with intracellular ATP content, cellular area, and circularity. Growth and morphology descriptors are functions that change on large timescales (hours) and provide indications about the general state of cells. ATP is a key intracellular parameter and energy currency that functions as substrate, activator, and inhibitor in many metabolic networks [47]. Its levels change within seconds of varying conditions (Additional file 2: Fig S3).

**Fig. 3:**
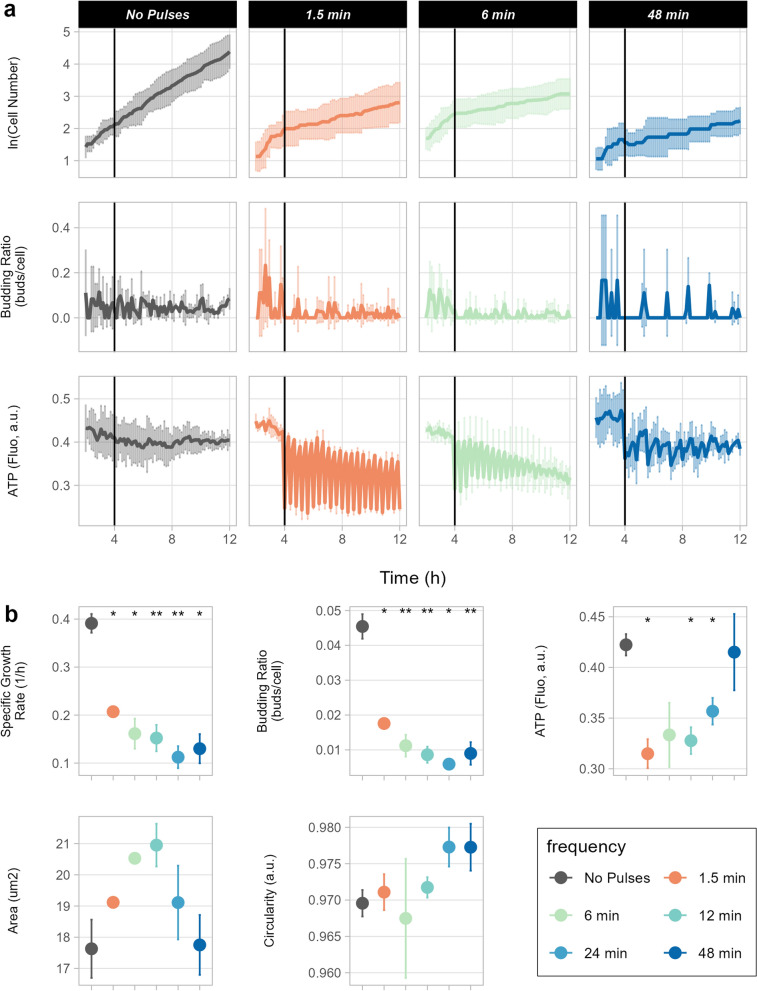
Population response and performance in dynamic feast-starvation oscillations. Error bars represent the standard deviation of triplicates (three chambers). (**a**) Line plots showing growth curve, budding ratio, and intracellular ATP level over the course of 12 h. The black vertical line indicates the beginning of glucose oscillation after 4 h of cultivation. Full 24-h screening data are available in Additional file 2: Figure S1. (**b**) Average specific growth rate, budding ratio, intracellular ATP level, area, and circularity of cells exposed to feast-starvation oscillations. Student’s t-test was performed to assess statistical differences between each feast-starvation oscillation frequency and the control condition (constant feast); *p ≤ 0.05, **p ≤ 0.01

Yeast cells exhibited lower growth in an oscillating environment (specific growth rates < 0.21 h^−1^) compared to the control condition (specific growth rate of 0.39 h^−1^); whereas ATP levels varied according to the feast and starvation cycle (Fig. 3a and Additional file 2: Fig S4). Slow oscillations (i.e. 48 min) triggered spikes in the budding rate during feast conditions which can be explained by lack of replication during starvation. Instead, shorter oscillations caused a more even distribution of budding events. Even though all cells were exposed to the same total feast or starvation time, the decrease in specific growth rate was frequency-dependent, dropping to a minimum of 0.11 h^−1^ with 24 min oscillations (Fig. 3b and Additional file 2: Fig S5). Frequency dependence of average specific growth rates in dynamic glucose environments has been reported also for *E. coli* [26].

Cell morphology was also dependent on frequency, as cells tended to become rounder and smaller with slower oscillations (Fig. 3b and Additional file 2: Fig S5). This phenomenon might correlate with optimisation of the surface-to-volume ratio and, hence, maximal nutrient uptake [48]. Such a hypothesis is supported by the observed increase in average ATP levels in cells exposed to longer oscillation frequencies (Fig. 3b and Additional file 2: Fig S5). The small cell size in the control condition can be correlated to the fast replication of cells. Cell circularity varied substantially with 1.5 and 6 min oscillations, owing to a shift towards pseudohyphal growth (Additional file 2: Fig S2). Pseudohyphal growth is a known response to nitrogen starvation and stress [49]. As shown by these examples, morphological changes induced by nutrient dynamics can be attained successfully in the proposed dMSCC setup.

**Fig. 4:**
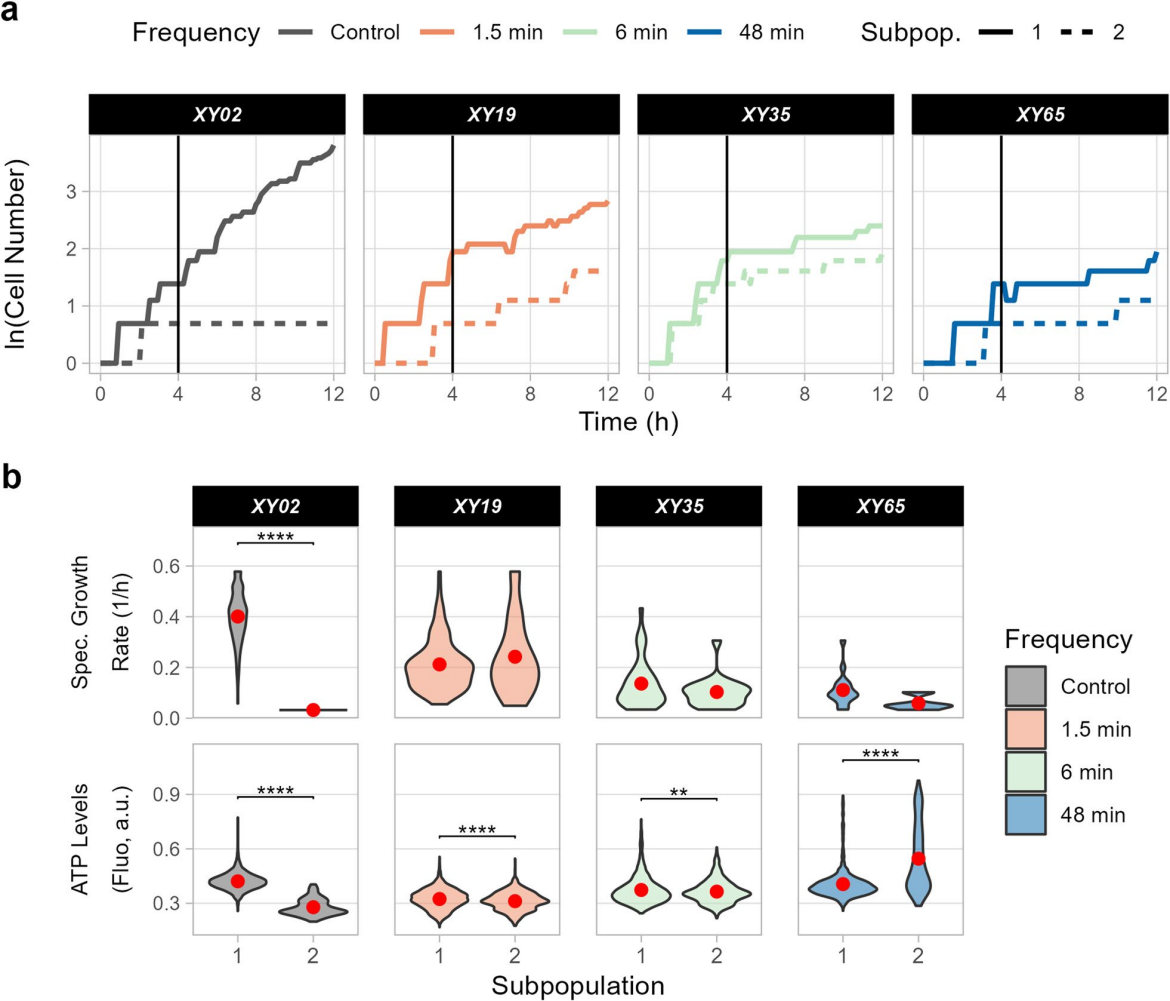
Subpopulation analysis in dMSCC. Subpopulations formed from the cells present in a chamber at the beginning of cultivation. Four chambers were analysed (XY02, XY19, XY35, and XY65), each characterised by a different oscillation frequency (control, 1.5, 6, and 48 min). A full overview of all chambers is provided in Additional file 2: Figure S8. (**a**) Growth curves of each subpopulation. (**b**) Violin plots highlighting the performance of single cells within the two subpopulations. Red dots denote the mean performance across all cells. Student’s t-test was performed to assess differences between the two subpopulations in each chamber; **p ≤ 0.01, ***p ≤ 0.001, and ****p ≤ 0.0001

Live-cell imaging extended beyond population-averaged measurements and achieved subpopulation resolution (Fig. 4). As the data are time-resolved, it is also possible to compare performance across ATP levels in starvation vs feast conditions (Additional file 2: Fig S6). Single-cell tracking enables the reconstruction of lineages for individual cells. As chambers were inoculated with one to three cells each, lineage reconstruction allowed the comparison of subpopulations derived from each initial cell (Fig. 4 and Additional file 2: Figs S7, S8). In some cases, a different growth behaviour across subpopulations (Fig. 4a and Additional file 2: Fig S7) or different specific growth rate and ATP levels within subpopulations were detected (Fig. 4b and Additional file 2: Fig S8). The rise of subpopulations is a common challenge in bioprocesses and only a few tools such as real-time flow cytometry enable its assessment at elevated temporal resolution [22, 50], further highlighting the potential of the proposed setup.

### Quantification and comparison of robustness in time for populations and single cells

**Fig. 5:**
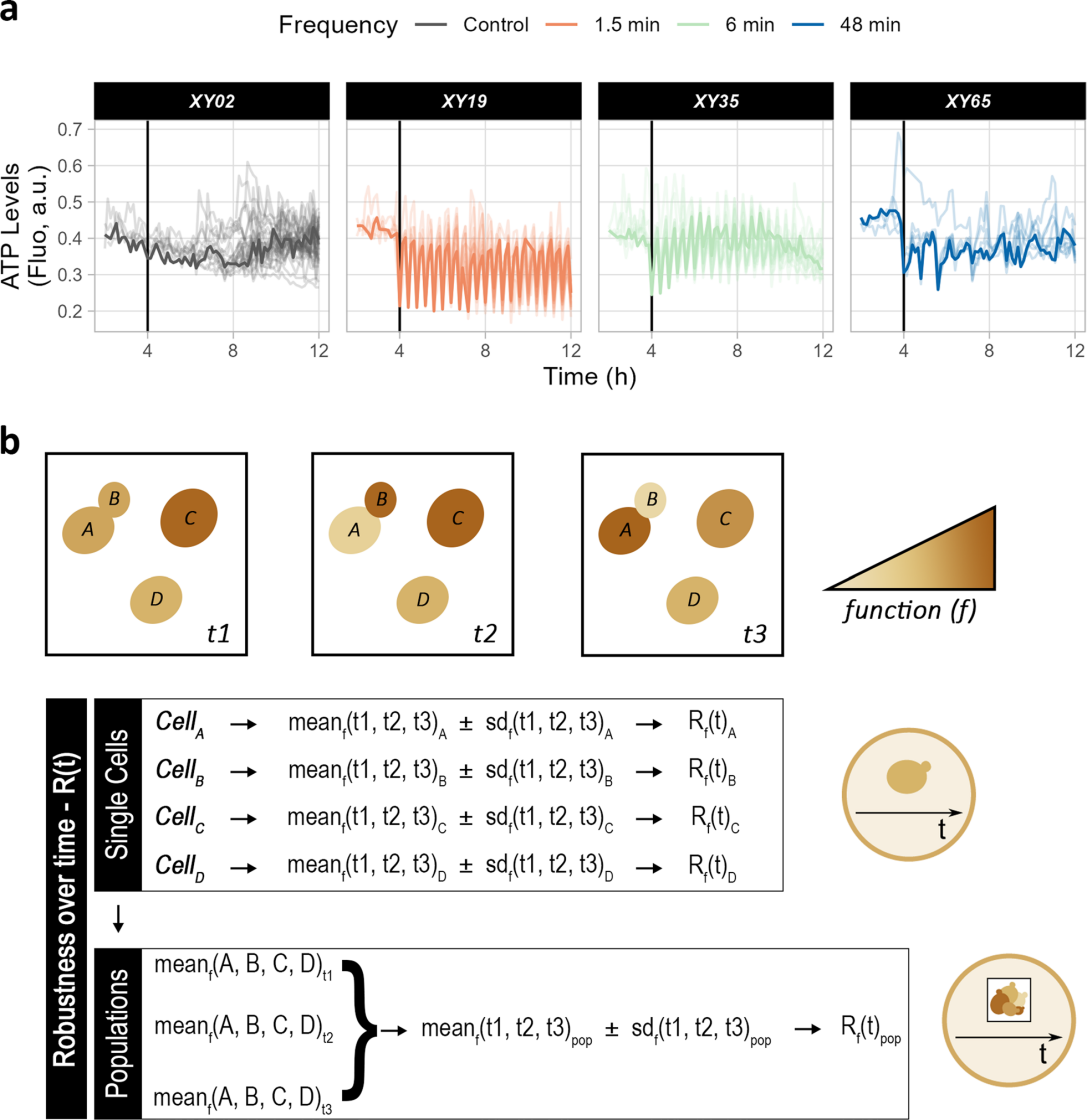
Computing robustness over time. (**a**) Representation of ATP levels over time for individual cells during the course of the first 12 h of cultivation. Each line represents a single cell. A random cell has been highlighted in each chamber – XY02 (control), XY19 (1.5 min), XY35 (6 min), and XY65 (48 min). (**b**) Graphical overview of R(t) at both population (chamber) and single-cell levels. Elevated R(t) values denote a stable function over time. At the single-cell level, mean and standard deviation of a function (e.g. ATP) in each individual cell (A, B, C, D) for all points in time (t1, t2, t3) are computed and used to derive R(t). At the chamber level, for each condition (oscillation frequency) and replicate (chamber), the average of a function for all cells at each time point is calculated and then used to compute the averaged function in time. The latter serves to calculate the mean and standard deviation of that function over time

Bioprocesses can benefit from stable product formation during microbial cultivation [7, 18, 50]. This is essential in continuous cultures, but it also ensures that microorganisms withstand stochastic perturbations [8]. The dMSCC setup allows the timely study of functions at single-cell resolution [23, 24, 50]. For instance, the monitoring of ATP levels in individual cells over time revealed large differences among cells and conditions (Fig. 5a). Therefore, the dMSCC setup was combined with a previously proposed robustness quantification method [9] (Eq. 1) to measure the stability of cellular functions over time, R(t), for each feast-starvation oscillation frequency. Elevated R(t) values indicate strong stability over time, while low values are observed when a function is more dispersed with respect to its mean. According to Eq. 1, R(t) changes upon addition of more replicates or conditions, making it a relative and not an absolute term.

**Fig. 6:**
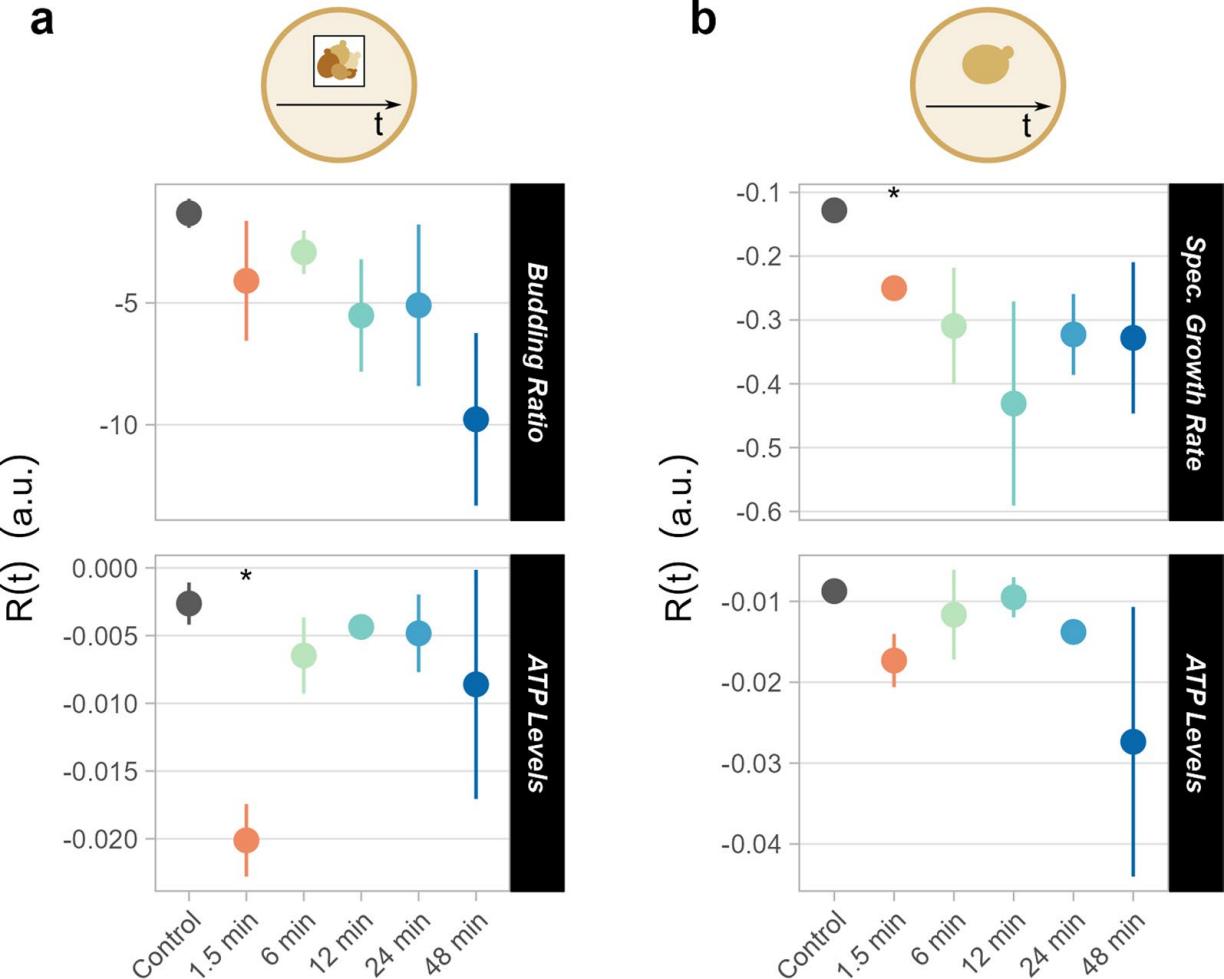
Robustness over time for growth functions and ATP levels. Representation of R(t) at the chamber (**a**) and single-cell (**b**) level for intracellular ATP and budding ratio/specific growth rate for all oscillation frequencies tested. Standard deviations represent the distribution across triplicates (chambers). For data about functions and distribution of single cells, refer to Additional file 2: Figure S9. *p ≤ 0.05

R(t) for the selected functions was computed both at the population (i.e. chamber) and single-cell levels (Fig. 5b). At the population level, the average performance for a function at each time point is computed, after which R(t) is generated based on the averaged measurement over time, with all time points contributing equally. Information describing the distribution of performance for a function at each time point is lost because of averaged measurements, thus masking single-cell behaviour. In contrast, at the single-cell level, R(t) is computed based on the mean performance of individual cells over time. As all cells contribute equally, time points with more cells may be overrepresented. The two R(t) values are, therefore, not expected to be equal, as confirmed for R(t) of ATP levels (Fig. 6), cell area, and circularity (Additional file 2: Fig S9).

R(t) was computed for growth descriptors (specific growth rate and budding ratio, see Methods), intracellular ATP, and morphology descriptors (Fig. 6 and Additional file 2: Fig S9). On the one hand, at population level, R(t) of the budding ratio decreased with slower oscillations due to longer stalls in replication (Fig. 6a). On the other hand, at single-cell level, R(t) of the specific growth rate was lowest at 12 min oscillations (Fig. 6b). This discrepancy confirmed the different trends for R(t) in populations vs single cells. Intracellular ATP levels displayed comparable trends in R(t) at chamber and single-cell levels between 1.5 and 24 min oscillations (Fig. 6 and Additional file 2: Fig S9), but differed substantially with 48-min oscillations, whereby R(t) was much lower for single cells. For morphology descriptors, the lowest R(t) values were associated with oscillations of 6 and 12 min, while the highest with those of 24 and 48 min (Additional file 2: Fig S9). The budding ratio and ATP levels became less stable over time, when exposed to starvation as opposed to feast conditions (Additional file 2: Figs S10, S11). Even though information on single-cell behaviour was lost in population-averaged data, R(t) often showed a similar trend at both levels, pointing to the usefulness of both approaches.

### Robustness quantification as a tool to describe population heterogeneity

**Fig. 7:**
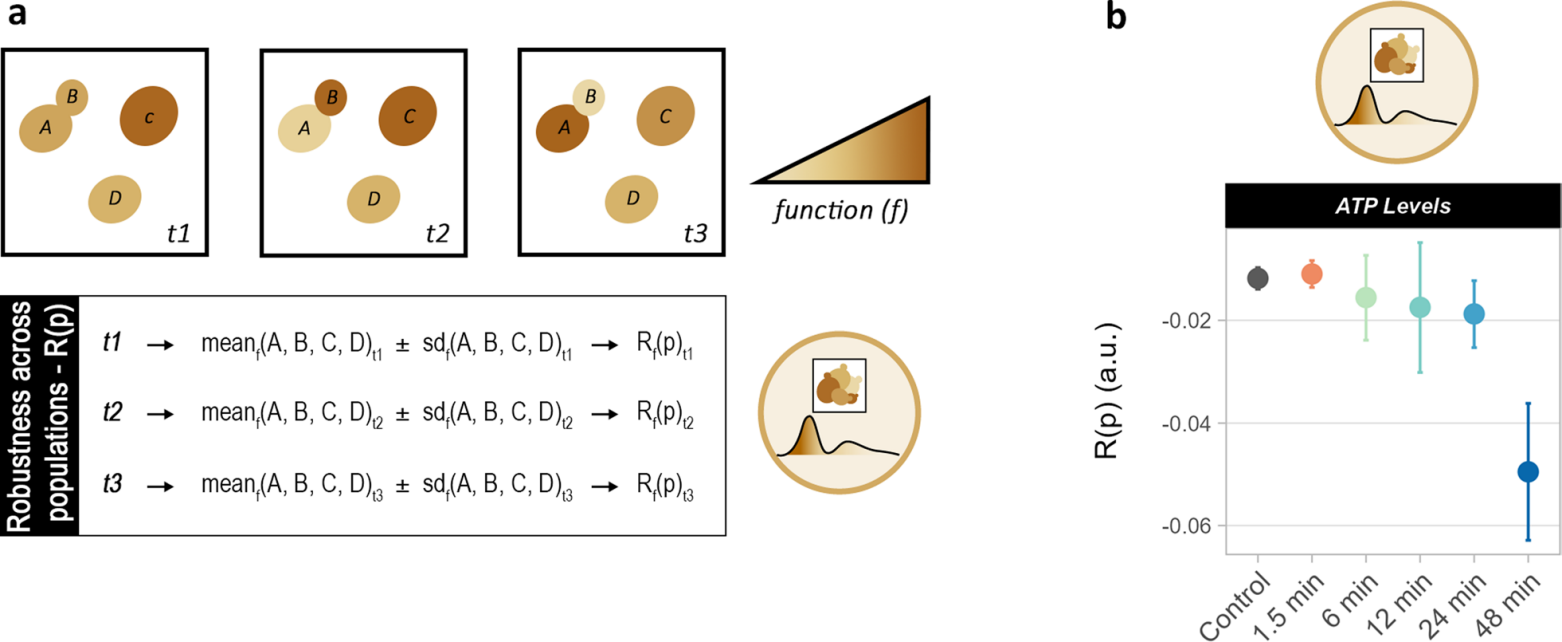
Robustness across populations as a description of population heterogeneity. (**a**) Visual representation of R(p) computation. R(p) denotes how homogeneous a function is within the same population at a single time point (t1, t2 or t3). Elevated R(p) describes a homogeneous function within the population, low R(p) a heterogeneous function. Here, the mean and standard deviation of a function among all cells (A, B, C, D) at each time point (t1, t2, t3) were calculated and used to compute R(p) for each time point. (**b**) R(p) of intracellular ATP levels for all tested oscillation frequencies. Standard deviations represent the distribution across triplicates (chambers). See Additional file 2: Figure S12 for all functions and distribution of R(p) at each time point (in violin plots)

R(t) was useful to describe the degree to which a function fluctuated or changed over time. However, information about the distribution of the function within the cell population at each time point was lost due to population-averaged data. Hence, the same robustness quantification method was applied to estimate the degree of population homogeneity, R(p) [11]. A population with elevated R(p) displays a homogeneous performance for a function across all cells at a given time point. Conversely, a low R(p) indicates greater population heterogeneity for that function (Fig. 7a).

In the present study, slower feast-starvation oscillations led to greater heterogeneity in intracellular ATP levels (Fig. 7b and Additional file 2: Fig S12), as manifested by a decrease in R(p) with longer oscillations. Populations showed elevated R(p) for ATP levels during exposure to starvation as opposed to feast conditions (Additional file 2: Figs S10a, S11). For cellular area and circularity, the lowest R(p) was observed with 6-min and 12-min-oscillations (Additional file 2: Fig S12a). Instead, at 6-min oscillations, pseudohyphal growth was triggered (Additional file 2: Fig S2), causing cells to present variable shapes and sizes. Overall, R(p) facilitates the comparison of population heterogeneity between different perturbation conditions by quantifying the distribution of performance for a population in a single value. This method marks a step forward towards understanding and studying such phenomena in both small-scale and large-scale fermentations.

## Discussion

### Interpretation in the context of bioprocess development

The design of modern bioprocess setups must consider strain performance and robustness in dynamic environments, as well as analysis of population heterogeneity at an early stage of development. So far, microbial selection and development have been based on consumption-based environmental changes and population-averaged measurements. As shown here, combining dMSCC with robustness quantification methods offers a high-throughput multi-level analysis of microbial cells in dynamic environments. Performance, its stability over time, and its distribution within a population can be measured at single-cell resolution. This enables the identification of stressful conditions and their effect on microbial cells.

**Fig. 8:**
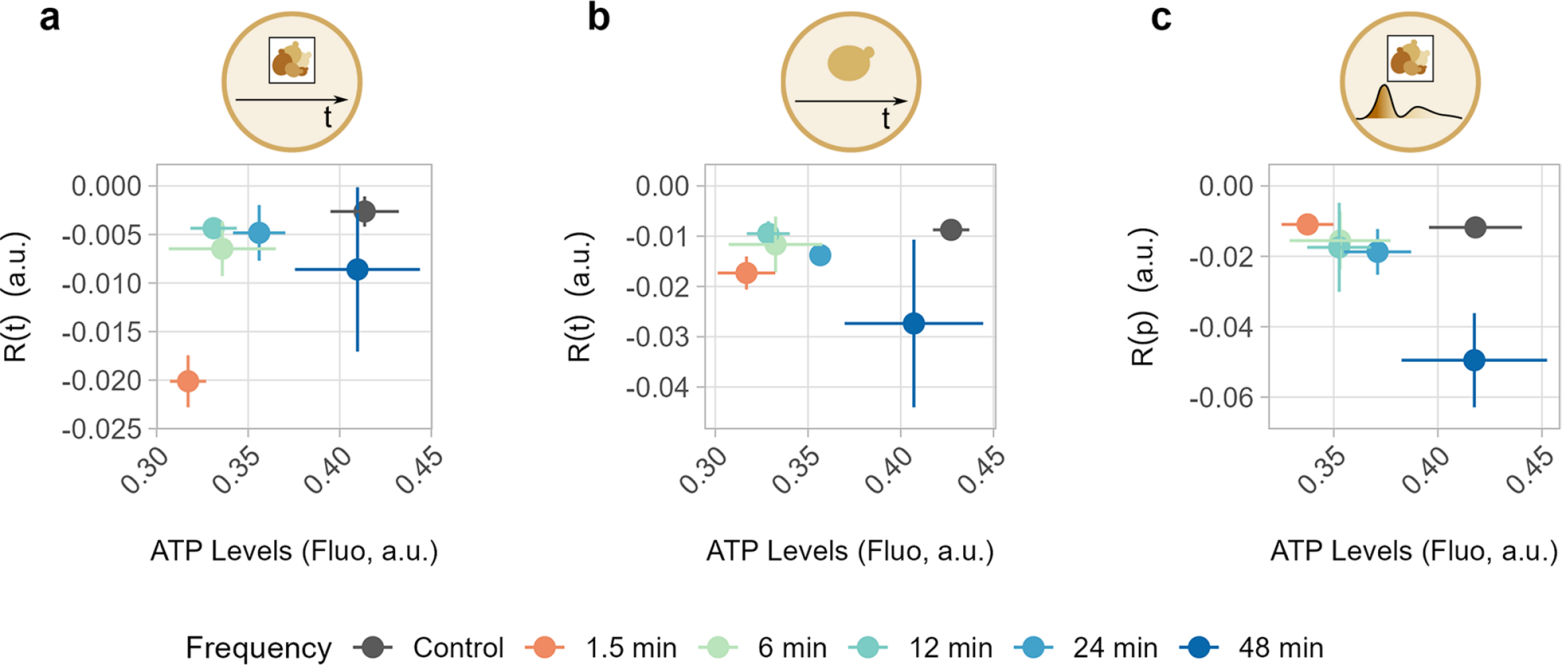
Robustness-vs-performance plots. Plots representing the correlation between performance (ATP level) and either R(p) (**a**) or R(t) at population (**b**) and single-cell level (**c**)

Optimal bioprocess productivity requires a stable and predictable microbial performance. Population heterogeneity lowers the predictability of a bioprocess and may affect its performance due to the emergence of a low-performing subpopulation [19, 21]. Changes in bioprocess conditions that may have a high impact on a microbial cell factory can be easily identified by investigating the relationship between either R(t) or R(p) and the respective performance of a function (Fig. 8 and Additional file 2: Fig S12). Taking ATP levels as an example, the highest performance was observed for cells subjected to 48 min oscillations, but this coincided with some of the lowest R(t) and R(p) values (Fig. 8). Cells in all other conditions had lower ATP levels, but higher R(t) and R(p). While those perturbations lowered the performance, stability was higher and therefore more predictable and reproducible. For other functions, such as growth, circularity or cell area, the optimal trade-off between performance and function stability varied widely (Additional file 2: Fig S12). Therefore, each combination of bioprocess condition and microorganism(s) should be evaluated separately to determine whether performance or robustness of a function is more important. For instance, a lower-performing but stable strain might be preferred for continuous cultures; whereas a higher-producing strain, which is less-stable over long periods of time, might be more suitable for batch cultures.

**Fig. 9:**
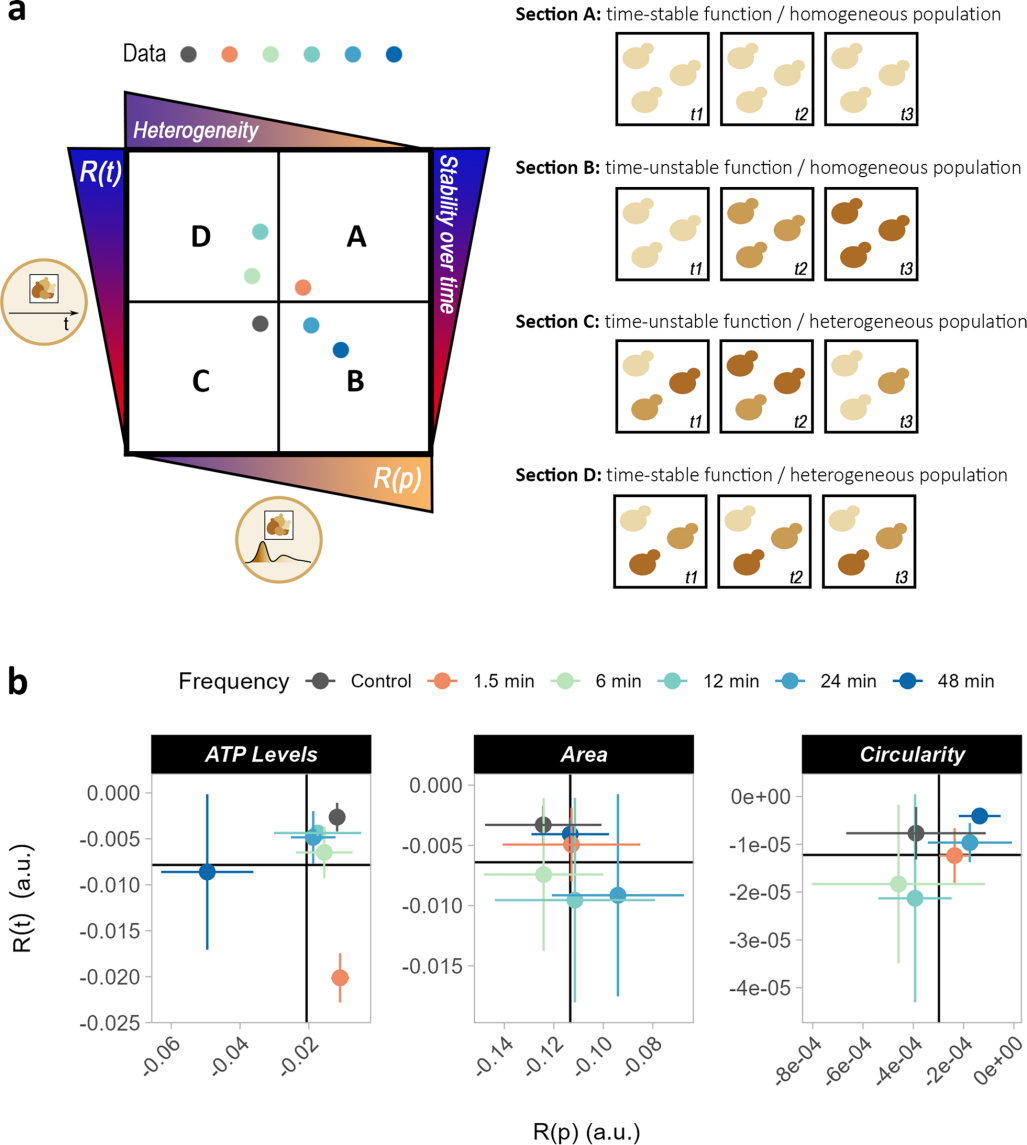
Robustness in time vs robustness across population. (**a**) Sections of the R(t) vs R(p) plot. In section A, the function is stable over time and homogenous in the population. In section B, the function is unstable over time but homogeneous within the population. In section C, the function is unstable over time and the population is heterogeneous. In section D, the function is stable over time, but heterogeneity is present in the population. (**b**) Data plots of R(t) vs R(p) for the following functions: relative ATP level, area, and circularity. The black cross indicates the mean R(t) and R(p) of each function for all oscillation frequencies. Standard deviation represents the distribution of replicates

Another interesting aspect is the correlation between R(t) and R(p), which might facilitate the identification of physiological responses to different conditions (Fig. 9a). For instance, oscillation frequencies that result in a stable function and no population heterogeneity will lie in section A, while both low R(t) and R(p) will define a condition as being in section C. Sections B and D are intermediate cases, in which cells are either stable over time or belong to a homogeneous population. For intracellular ATP levels (Fig. 9b), most oscillation frequencies fell in section A, indicating good stability over time and within the population. In contrast, cells subjected to 48-min oscillations fell in section C, which coincided with population heterogeneity and unstable ATP levels over time. Cells undergoing 1.5 min oscillations featured homogeneity and high instability of functions over time, thereby falling in section B. Finally, slower oscillations had a stabilizing effect on cell area and circularity over time and within a population (Fig. 9b). These considerations are of interest when comparing strains and their production performance in dynamic environments, particularly if the strains fall into sections B or D. In section B, strains display an unstable function over time but a homogeneous population, which would make them particularly sensitive to environmental fluctuations and, consequently, alter production. Therefore, efforts should be aimed at improving either the strains to withstand fluctuations or the reactor to maintain a more stable environment. Strains in section D might be composed of distinct subpopulations with different production abilities, but a stable performance over time. In this case, efforts should be directed towards improving the strains to avoid subpopulations characterised by different production abilities. Notably, these points do not consider strain performance, but only its stability.

So far, performance of desired functions has been the main criterion when selecting microorganisms for industrial applications. Robustness quantification in combination with dMSCC enables a more tailored approach, as different aspects of function stability in dynamic environments and possible population heterogeneity can be integrated into strain selection. The pipeline allows for easy identification of adverse conditions based on their influence on performance, R(t), and R(p). In future applications, the high experimental parallelisation and analysis depth offered by the proposed method will facilitate testing and comparison between strains.

### Towards downscaling of bioreactor gradients

Cell responses to environments that change within seconds or minutes have been largely overlooked when designing microbial cell factories, even though they are important in large-scale processes. Implementing a quantitative assessment of how stable a function is would improve strain and bioreactor design. In the present work, dMSCC was combined with robustness quantification to study growth, ATP levels, cell area, and morphology under feast-starvation oscillations in *S. cerevisiae* at population, subpopulation, and single-cell level. Such oscillations were symmetric and spanned from 1.5 to 48 min, thereby covering different biological timescales. A decrease in the specific growth rate but increase in ATP levels was observed with longer oscillation intervals. Furthermore, a change in cell morphology was observed, as pseudohyphal growth was triggered by glucose shifts every 1.5 and 6 min (Additional file 2: Fig S1). Cells subjected to 48 min oscillations exhibited the highest average ATP content, but the lowest stability over time and the highest population heterogeneity.

To increase the applicability of the pipeline for bioprocess development, the oscillations can be adapted to better represent large-scale heterogeneities. For example, glucose oscillations below 2 min are a reasonable choice, as they fall within the mixing time for 95% homogeneity in large-scale vessels [51]. Other scale-down approaches limited the residence time in starvation regimes to 2 min, followed by an asymmetric recovery time [17]. The amplitude (concentration range) of glucose oscillations can be easily adapted to resemble common large-scale conditions. A glucose concentration range of 0–25 mg/L has been reported for yeast fermentations [32, 52], while simulations used a range of 0–100 mg/L of glucose [53]. Experimental conditions can be guided also by lifeline analysis of broth heterogeneity, as is modern practice for scale-down bioreactor experiments [53, 54].

By assessing the correlation between robustness and performance or between R(t) and R(p) in representative glucose oscillations, it is possible to estimate effects of dynamic perturbations early in bioprocess development.

### Advantages and limitations of the pipeline

The dMSCC offered multiple advantages, such as excellent environmental control and manipulation, high parallelisation, and single-cell resolution, which enabled the tracking of individual cells and their reactions in time [24]. Nevertheless, some trade-offs were inevitable. The number of tested conditions, replicates, and applied biosensors limits temporal resolution as time is needed for image acquisition. Imaging rapid changes with a low temporal resolution, however, introduces aliasing effects. On the one hand, imaging frequency faster than the target oscillation offered a detailed overview of the fluctuations characterising the studied function (Additional file 2: Fig S3). On the other hand, imaging frequencies slower than the target oscillation (e.g. 1.5 and 6 min), provided a lower temporal resolution of the dynamics studied (Additional file 2: Fig S4). These considerations are valid for rapidly changing functions such as ATP levels, but do not apply to slow-adapting functions, such as specific growth rate or morphology. Furthermore, live-cell imaging in monolayer-growth chambers affects yeast cells through phototoxicity when applying fluorescent biosensors [45]. Cells perturbed by environmental stress were reported to be more susceptible to phototoxicity than unperturbed cells [55]. As the perceived light dose is a major determinant of the severity of phototoxicity, a trade-off arises between high temporal resolution, multiple biosensors, and fluorescence detection. Another factor is mechanical stress. Cells in monolayer-growth chambers are immobilised by trapping between PDMS and glass during cultivation and movement is only possible when culture expansion pushes cells outside the chamber. Such conditions exert compressive and tensile forces on the cells, which are absent from cells in suspension [56]. Arguably, other cultivation devices also introduce mechanical artefacts such as pumping. Importantly, dMSCC allows for a preliminary, high-throughput, lab-scale investigation of rapid changes, which could validate mathematical predictions and improve in silico metabolic models [54].

Increasing the experimental throughput requires automated data processing. Therefore, a semi-automated pipeline for image analysis in Fiji was developed herein, combining StarDist-2D for segmentation with TrackMate to track individual cells and their time-dependent functions and lineages. In previous microfluidics setups, only mother cells were monitored over time in single-cell traps [57] or they were grown in a small narrow chamber for easier bud identification [58]. Recently, other approaches to monitor cells have been developed [59], but the pipeline proposed in this study has the advantage of requiring no programming skills and easy adaptability to new developments or research goals. In particular, converting the pipeline from a Fiji macro to python language can fully automate the procedure, although tracks would still need to be checked and edited manually, as they might not be reliable in the case of rapid growth or low temporal resolution. Overall, this pipeline for image analysis was built to be easily customisable by the user, as well as to enable the integration of new and faster tools for segmentation and tracking. For example, if only population behaviour data are needed, it is possible to have a fully automated pipeline using only StarDist-2D, with no track checking and editing steps. Despite losing time-dependency information on single cells, R(p) provides an assessment of population heterogeneity (Additional file 3).

Here, we quantified robustness by applying a formula that identified stable functions across different conditions [9], including time and populations [11]. R(t) enabled the quantification of how much the desired functions were dispersed with respect to their mean during the experimental period. Such analysis is generally qualitative and unsuitable for a comparison of high-throughput data. R(t) is not able to differentiate between oscillating and steadily changing functions. However, other mathematical approaches, like derivatives or peak analysis, can be applied on the same data for more detailed analysis. Therefore, R(t) is a useful parameter to describe stable performances of strains in large-scale environments and estimate the success of strain engineering for higher stability. *E. coli* production rates and titres, estimated by eGFP-marked proteins, have been ameliorated by strengthening the cells’ performance in dynamic environments, partly as a result of reduced population heterogeneity [18]. Such improvement in stability over time could have been quantified using the approach proposed in this study to facilitate strain comparison.

Population heterogeneity is common during large-scale fermentation or long-term cultivations [19]. In the applied dMSCC system, a maximum of 35.000 yeast cells can be analysed when all monolayer-growth chambers in an oscillation structure are captured (six arrays × 23 chambers × 250 cells per chamber). Arguably, this population size is multiple magnitudes smaller than in any bioreactor. Rare events within populations (< 1/1000) can therefore not be captured. In bioprocesses, averaged yield, titers and rates are however determined by major subpopulations and not by rare events [21]. Such subpopulations can be detected within a population of 1000 cells, allowing for the application of dMSCC for analysis. By computing R(p) of a function in this work, it was possible to estimate population heterogeneity at each time point of the screening and, therefore, compare conditions and strains. Mathematical description of population heterogeneity using mean- and standard deviation-based methods is limited by the appearance of distinct subpopulations, which generate bi-modal or multi-modal distributions [60]. Nevertheless, if a heterogeneous population is detected, the same data can be used to determine the type of heterogeneity based on e.g. population entropy [50].

Furthermore, R(p) can be applied to other acquisition approaches such as real-time flow-cytometry, which can capture population heterogeneity in dynamic environments when coupled to a scale-down reactor [22]. R(p) evaluation in combination with dMSCC enables easy detection and comparison of heterogeneity for different conditions and strains.

The presented pipeline could facilitate the study of cell behaviour in a rapidly dynamic environment, but also assess robust microbial performance under gradients similar to those in large-scale fermentations. In this work, multiple timescales describing microbial reactions were covered. Moreover, the proposed pipeline, as well as the combination of robustness quantification and dMSCC, can be easily adapted to accommodate different setups and questions, including a more accurate imitation of large-scale heterogeneities. Overall, the benefits of robustness quantification in dynamic environments using dMSCC rest mainly on experimental throughput, different levels of resolution, a multitude of possible applications, as well as comparability between strains, conditions, and experiments. These benefits can facilitate and accelerate bioprocess development and strain optimisation for new and robust bioprocesses.

## Conclusion

Here, we offer a pipeline that implements robustness quantification to rapid environmental changes (seconds to minutes) in response to nutrient availability. The pipeline served to investigate the performance and robustness of the following functions: growth, ATP levels, and morphology of yeast cells. Using dMSCC to simulate a dynamic environment, yeast cells were subjected to feast-starvation cycles ranging between 1.5 and 48 min. We believe the proposed method is valuable not only for answering basic questions about strain performance, but also to understand effects of the dynamics that cells are subjected to during large-scale production. In fact, the combination of dMSCC and robustness quantification might help bridge the gap between lab- and large-scale settings, allowing for a more reliable characterisation of microbial strains already during bioprocess development or improvement. Including robustness quantification in the analysis might reveal different trade-offs with respect to performance. Here, it was used to assess the stability of various functions over time and their heterogeneity within the cell population, as well as at subpopulation and single-cell level.

### Supplementary Information

**Additional file 1: **Video comparing growth of one replicate (chamber) for each feast-starvation oscillation frequency, along with the control condition (constant feast).

**Additional file 2: Figure S1.** Microscopy images from the dMSCC setup. Microscopy images showing one replicate (chamber) for each feast-starvation oscillation frequency (from 1.5 to 48 min) and constant feast conditions (control). Images were taken at 4 h (a) and 24 h (b) from the onset of cultivation. Colour denotes the ATP content inside each cell, with green indicating low and blue high levels. The scale bar is 15 µm. **Figure S2.** Pseudohyphal growth in the dMSCC setup. Microscopy images showing one replicate (chamber) for each feast-starvation oscillation frequency (from 1.5 to 48 min) and constant feast conditions (control). Images were taken at 20 h from the onset of cultivation. Colour denotes the ATP content inside each cell, with green indicating low and blue high levels. In cells subjected to oscillations of 1.5 and 6 min, pseudohyphal growth was observed, as shown by red arrows in enlarged images. The scale bar is 15 µm. **Figure S3.** High temporal-resolution imaging of yeast in feast-starvation oscillations. Line plots indicate ATP levels in cells exposed for 2 h to oscillating feast-glucose conditions (1.5 and 6 min) or constant feast conditions (control). ATP levels were monitored using the fluorescent biosensor QUEEN-2m. Images were taken every 17 s. Each line plot represents a single replicate (chamber, named as “XY”). The standard deviation corresponds to the distribution of ATP levels across the cell population at each time point in each chamber. **Figure S4.** Overview of cellular functions throughout the screening period. Line plots for functions (budding ratio, relative ATP concentration, cell area, and cell circularity) of yeast cells subjected to feast-starvation oscillations. Error bars denote the standard deviation within the population-averaged performance of triplicates (three chambers). Line plots for individual chambers can be found in Additional File 3. **Figure S5.** Distribution of performance data. Distribution of performance data relative to cellular functions and based on single-cell data except for budding rate, which was computed at the chamber level. Violin plots encompass distinct time points and red dots represent the mean across all cells/time points in that condition. All triplicates (three chambers) were considered together. Violin plots for each individual replicate (chamber) are found in Additional File 3. Student’s t-test was performed to assess statistical differences between each feast-starvation oscillation frequency and the control condition (constant feast); ****p ≤ 0.0001. **Figure S6.** Comparison of performance with respect to condition. Performance data for budding ratio and ATP levels have been divided based on whether they were taken during a feast or starvation condition. “TOT” refers to data for the whole screening. Dispersion of the data corresponds to the standard deviation across triplicates (three chambers). **Figure S7.** Growth line plots for distinct cell subpopulations. For each replicate (i.e. chamber named “XY”), different subpopulations formed during the cultivation period are shown. Each line represents the subpopulation originating from an individual cell present at the beginning of the cultivation. Each chamber was inoculated with 1–4 cells. **Figure S8.** Performance distribution of functions across subpopulation. For each chamber (named “XY”), subpopulations originating from an initial inoculum of 1–4 cells are shown. Violin plots present the single-cell performance for the following functions: specific growth rate, ATP levels, area, and circularity. The red dot in each violin plot represents the mean performance of that subpopulation. ANOVA was performed for each chamber to determine if the mean performances of subpopulations differed from one another; *p ≤ 0.05, **p ≤ 0.01, ***p ≤ 0.001, and ****p ≤ 0.0001. **Figure S9.** Robustness over time. Robustness over time, R(t), denotes how stable a function is over time. Elevated R(t) values are associated with stable functions over time, while low R(t) values with unstable ones. R(t) was computed for the desired functions at the population (a) and single-cell (b) levels. The standard deviation refers to the distribution of triplicates (three chambers). (c) Distribution of R(t) for each individual cell. Red dots represent the mean R(t) for each condition. Student’s t-test was used to evaluate the statistical difference of single-cell R(t) between each condition and the control; *p ≤ 0.05, **p ≤ 0.01, ***p ≤ 0.001, and ****p ≤ 0.0001. **Figure S10.** Comparison of robustness with respect to condition. Robustness data for ATP levels (a) and budding ratio (b) are categorised based on whether they were taken during a feast or starvation condition. “TOT” refers to data for the whole screening. Dispersion of the data refers to the standard deviation across triplicates (three chambers). Robustness quantification was used to compute robustness over time, R(t), at population (panel a, top, and panel b) and single-cell levels (panel a, middle), as well as robustness across populations (panel a, bottom), R(p), to assess the stability of a function with respect to population heterogeneity. **Figure S11.** Violin plots of robustness with respect to pulse. (a) Distribution of single-cell-level robustness over time for ATP content with respect to pulse (feast or starvation). “TOT” refers to data for the whole screening. Red dots represent the mean R(t) across all cells at each pulse. (b) Distribution of robustness across populations for ATP levels with respect to pulse (feast or starvation). “TOT” refers to data for the whole screening. Red dots represent the mean R(p) across all time points for each pulse. Student’s t-test was used to evaluate the statistical difference of either R(t) or R(p) between starvation and feast pulses; *p ≤ 0.05, **p ≤ 0.01, ***p ≤ 0.001, and ****p ≤ 0.0001. **Figure S12.** Robustness across populations. Robustness across populations, R(p), denotes how stable a function is across a population at each time point. Elevated R(p) values are associated with homogeneous populations, while low R(p) values with heterogeneous ones. R(p) was computed for the desired functions (ATP levels, area, and circularity). (a) The standard deviation refers to the distribution across triplicates (three chambers). (b) Violin plots denote the distribution of R(p) for each time point. Each violin plot considers triplicates (three chambers) together. Data pertaining to each chamber are presented in Additional File 3. Red dots represent the mean R(p) across all time points for each condition. Student’s t-test was used to evaluate the statistical difference between each condition and the control; *p ≤ 0.05, **p ≤ 0.01, ***p ≤ 0.001, and ****p ≤ 0.0001. **Figure S13.** Robustness vs performance plots. Correlation between performance and robustness over time at the population level (a) or robustness over time at the single-cell level (b), as well as robustness across populations (c) for selected cellular functions.

**Additional file 3: Figure S1.** Line plots for ATP levels over time. Line plots for ATP levels measured throughout the screening (24 h) in each chamber (named “XY”). Error bars refer to the standard deviation of the function at each time point across the entire cell population. The vertical line at 4 h identifies the beginning of the starvation-oscillation period. **Figure S2.** Line plots for growth curves. Line plots for growth curves measured throughout the screening (24 h) for each chamber (named “XY”). The vertical line at 4 h identifies the beginning of the starvation-oscillation period. **Figure S3.** Line plots for budding ratio over time. Line plots for the budding ratio measured throughout the screening (24 h) for each chamber (named “XY”). The vertical line at 4 h identifies the beginning of the starvation-oscillation period. **Figure S4.** Line plots for area over time. Line plots for the cellular area measured throughout the screening (24 h) for each chamber (named “XY”). Error bars refer to the standard deviation of the function at each time point across the whole cell population. The vertical line at 4 h identifies the beginning of the starvation-oscillation period. **Figure S5.** Line plots for circularity over time. Line plots for cellular circularity measured throughout the screening (24 h) for each chamber (named “XY”). Error bars refer to the standard deviation of the function at each time point across the whole cell population. The vertical line at 4 h identifies the beginning of the starvation-oscillation period. **Figure S6.** Violin plots showing the performance of individual chambers. Violin plots showing the distribution of performance data in individual chambers (named “XY”). “Merged” refers to the chamber triplicates considered altogether. The red dot denotes the mean across all cells in that chamber. The dashed horizontal line is the mean of the merged chambers. **Figure S7.** Violin plots showing robustness over time of individual chambers. Violin plots showing the distribution of data for robustness over time at single-cell level in individual chambers (named “XY”). “Merged” refers to the chamber triplicates considered altogether. The red dot denotes the mean across all cells in that chamber. The dashed horizontal line is the mean of the merged chambers. **Figure S8.** Violin plots showing robustness across populations in individual chambers. Violin plots showing the distribution of data for robustness across populations in individual chambers (named “XY”). “Merged” refers to the chamber triplicates considered altogether. The red dot denotes the mean across all cells in that chamber. The dashed horizontal line is the mean of the merged chambers.

## Data Availability

Data, R scripts, and the Fiji macro used in this study are available via GitHub (https://github.com/lucatorep/Robustness_Microfluidics). In each script and macro, description of lines and code section has been carefully curated. The live-cell imaging dataset is available from the corresponding author upon reasonable request.

## Abbreviations

dMSCC: Dynamic microfluidic single-cell cultivation
R(t): Robustness over time
R(p): Robustness over population
