# Supplementary material for: Quantifying microbial robustness in dynamic environments using microfluidic single-cell cultivation
Source: Microb Cell Fact. 2024 Feb 9;23:44. doi: 10.1186/s12934-024-02318-z (PMC10854032; doi:10.1186/s12934-024-02318-z)
Supplement: Supplementary file 2 — Additional file 2: Figure S1. Microscopy images from the dMSCC setup. Microscopy images showing one replicate (chamber) for each feast-starvation oscillation frequency (from 1.5 to 48 min) and constant feast conditions (control). Images were taken at 4 h (a) and 24 h (b) from the onset of cultivation. Colour denotes the ATP content inside each cell, with green indicating low and blue high levels. The scale bar is 15 µm. Figure S2. Pseudohyphal growth in the dMSCC setup. Microscopy images showing one replicate (chamber) for each feast-starvation oscillation frequency (from 1.5 to 48 min) and constant feast conditions (control). Images were taken at 20 h from the onset of cultivation. Colour denotes the ATP content inside each cell, with green indicating low and blue high levels. In cells subjected to oscillations of 1.5 and 6 min, pseudohyphal growth was observed, as shown by red arrows in enlarged images. The scale bar is 15 µm. Figure S3. High temporal-resolution imaging of yeast in feast-starvation oscillations. Line plots indicate ATP levels in cells exposed for 2 h to oscillating feast-glucose conditions (1.5 and 6 min) or constant feast conditions (control). ATP levels were monitored using the fluorescent biosensor QUEEN-2m. Images were taken every 17 s. Each line plot represents a single replicate (chamber, named as “XY”). The standard deviation corresponds to the distribution of ATP levels across the cell population at each time point in each chamber. Figure S4. Overview of cellular functions throughout the screening period. Line plots for functions (budding ratio, relative ATP concentration, cell area, and cell circularity) of yeast cells subjected to feast-starvation oscillations. Error bars denote the standard deviation within the population-averaged performance of triplicates (three chambers). Line plots for individual chambers can be found in Additional File 3. Figure S5. Distribution of performance data. Distribution of performance data relative to cell [file 12934_2024_2318_MOESM2_ESM.pdf]

Additional File 2

Quantifying microbial robustness in dynamic environments using microfluidic single-cell cultivation

Luisa Blöbaum[§1,2], Luca Torello Pianale[§3], Lisbeth Olsson[3*], Alexander Grünberger*[1,4]

[1]Multiscale Bioengineering, Technical Faculty, Bielefeld University, Bielefeld, Germany

[2]CeBiTec, Bielefeld University, Bielefeld, Germany

[3]Industrial Biotechnology Division, Department of Life Sciences, Chalmers University of Technology, Gothenburg, Sweden

[4]Microsystems in Bioprocess Engineering, Institute of Process Engineering in Life Sciences, Karlsruhe Institute of Technology, Karlsruhe, Germany

[§]Equal contribution

*Correspondence: Alexander Grünberger (alexander.gruenberger@kit.edu).

Supplementary Information

The “Supplementary Figures” section includes:

- Microscopy images from the dMSCC setup (Supplementary Figures S1–S2).
- High temporal-resolution imaging of yeast (Supplementary Figure S3).
- Overview of cellular functions throughout the screening period (Supplementary Figure S4).
- Distribution of performance data (Supplementary Figure S5).
- Comparison of performance with respect to pulse (Supplementary Figure S6).
- Growth line plots for distinct cell subpopulations (Supplementary Figure S7).
- Performance distribution of functions across subpopulations (Supplementary Figure S8).
- Robustness over time (Supplementary Figure S9).
- Comparisons of robustness with respect to pulse (Supplementary Figures S10–S11).
- Robustness across populations (Supplementary Figure S12).
- Robustness vs performance plots (Supplementary Figure S13).

Supplementary Figures

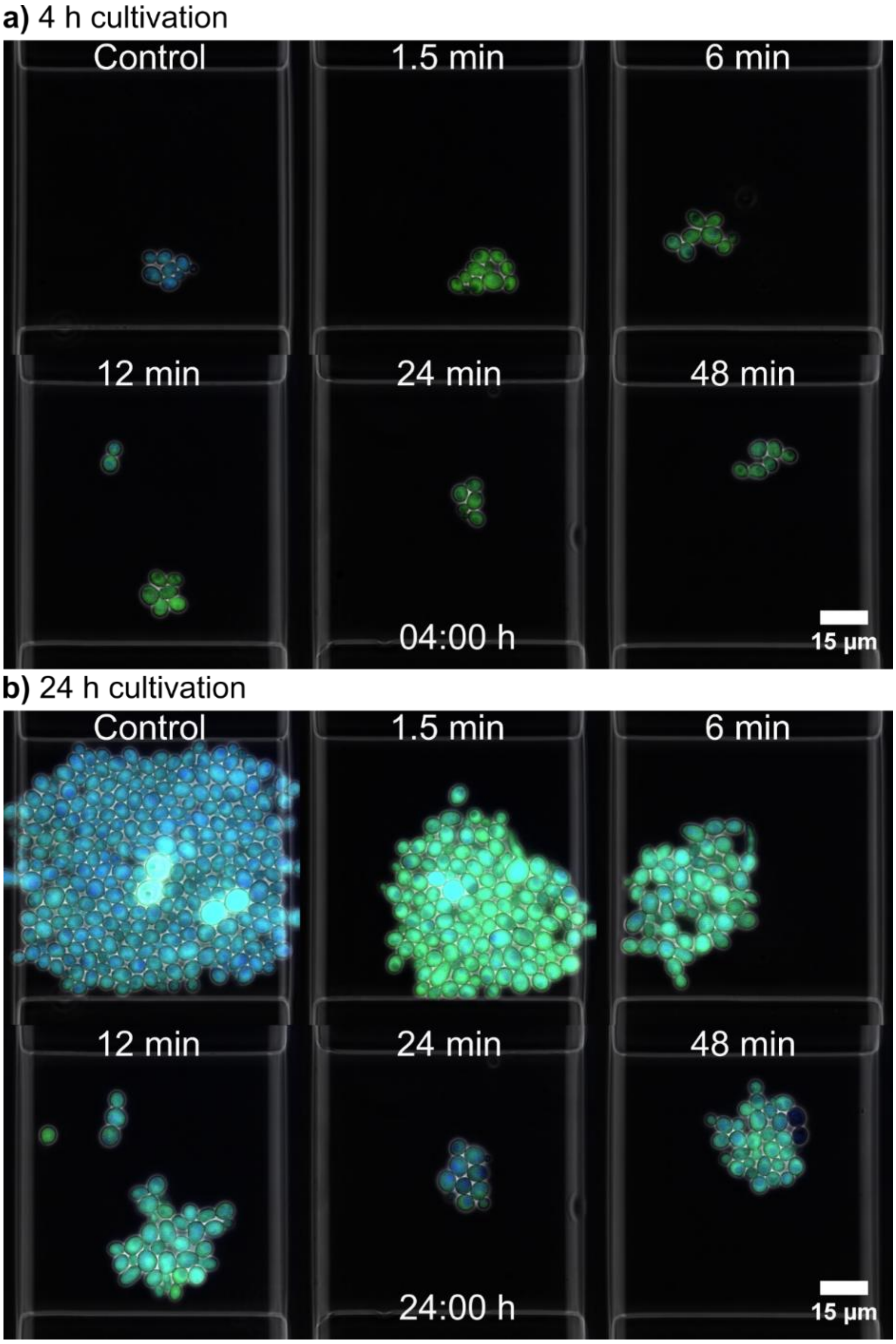

Supplementary Figure S1. Microscopy images from the dMSCC setup. Microscopy images showing one replicate (chamber) for each feast-starvation oscillation frequency (from 1.5 to 48 min) and constant feast conditions (control). Images were taken at 4 h (a) and 24 h (b) from the onset of cultivation. Colour denotes the ATP content inside each cell, with green indicating low and blue high levels. The scale bar is 15 µm.

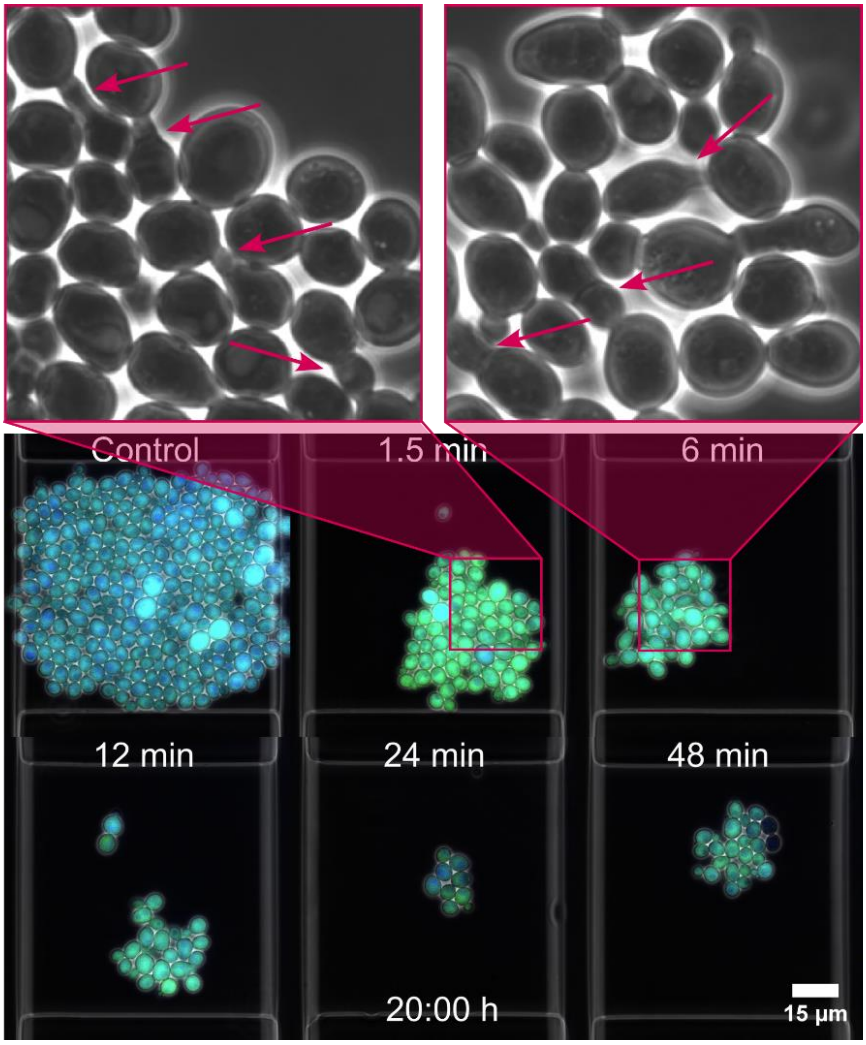

Supplementary Figure S2. Pseudohyphal growth in the dMSCC setup. Microscopy images showing one replicate (chamber) for each feast-starvation oscillation frequency (from 1.5 to 48 min) and constant feast conditions (control). Images were taken at 20 h from the onset of cultivation. Colour denotes the ATP content inside each cell, with green indicating low and blue high levels. In cells subjected to oscillations of 1.5 and 6 min, pseudohyphal growth was observed, as shown by red arrows in enlarged images. The scale bar is 15 µm.

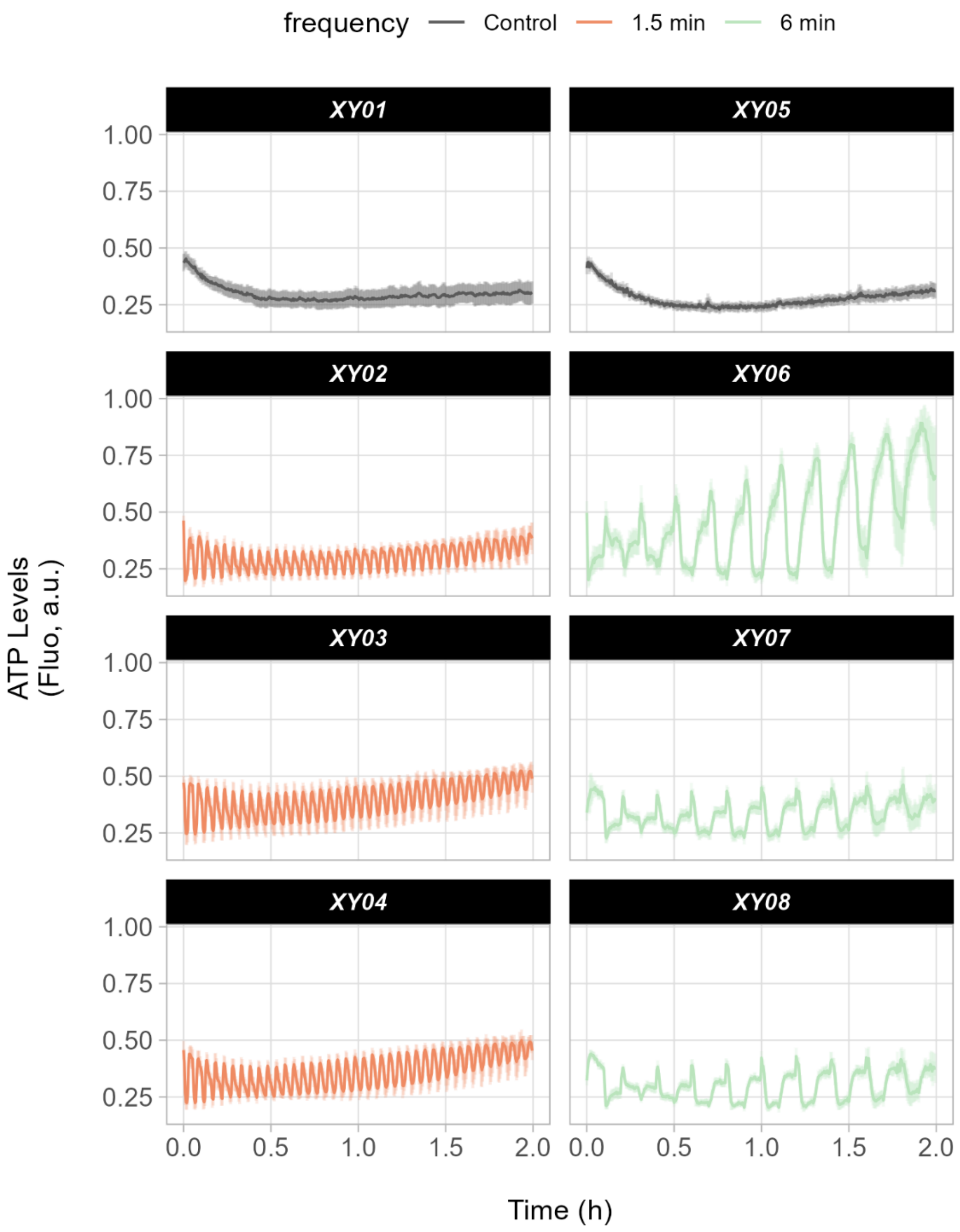

Supplementary Figure S3. High temporal-resolution imaging of yeast in feast-starvation oscillations. Line plots indicate ATP levels in cells exposed for 2 h to oscillating feast-glucose conditions (1.5 and 6 min) or constant feast conditions (control). ATP levels were monitored using the fluorescent biosensor QUEEN-2m. Images were taken every 17 s. Each line plot represents a single replicate (chamber, named as “XY”). The standard deviation corresponds to the distribution of ATP levels across the cell population at each time point in each chamber.

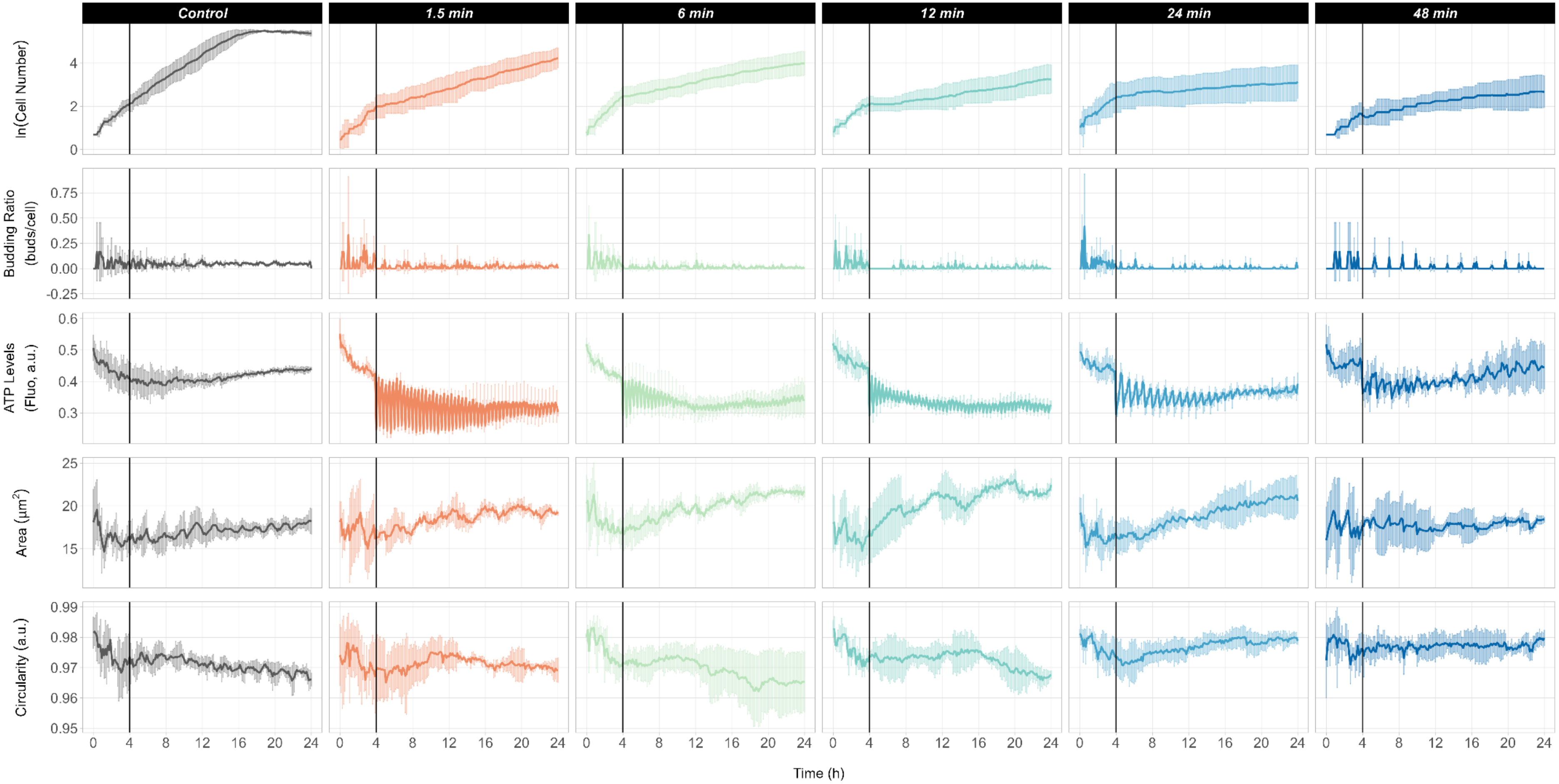

Supplementary Figure S4. Overview of cellular functions throughout the screening period. Line plots for functions (budding ratio, relative ATP concentration, cell area, and cell circularity) of yeast cells subjected to feast-starvation oscillations. Error bars denote the standard deviation within the population-averaged performance of triplicates (three chambers). Line plots for individual chambers can be found in Additional File 3.

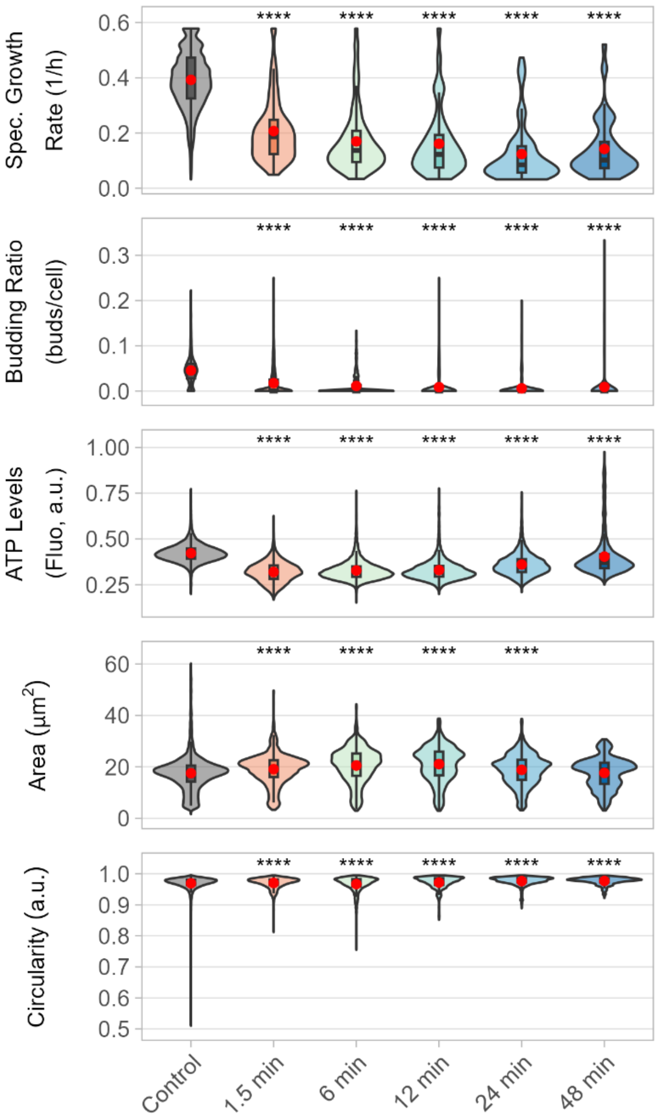

Supplementary Figure S5. Distribution of performance data. Distribution of performance data relative to cellular functions and based on single-cell data except for budding rate, which was computed at the chamber level. Violin plots encompass distinct time points and red dots represent the mean across all cells/time points in that condition. All triplicates (three chambers) were considered together. Violin plots for each individual replicate (chamber) are found in Additional File 3. Student's *t*-test was performed to assess statistical differences between each feast-starvation oscillation frequency and the control condition (constant feast); ****p ≤ 0.0001.

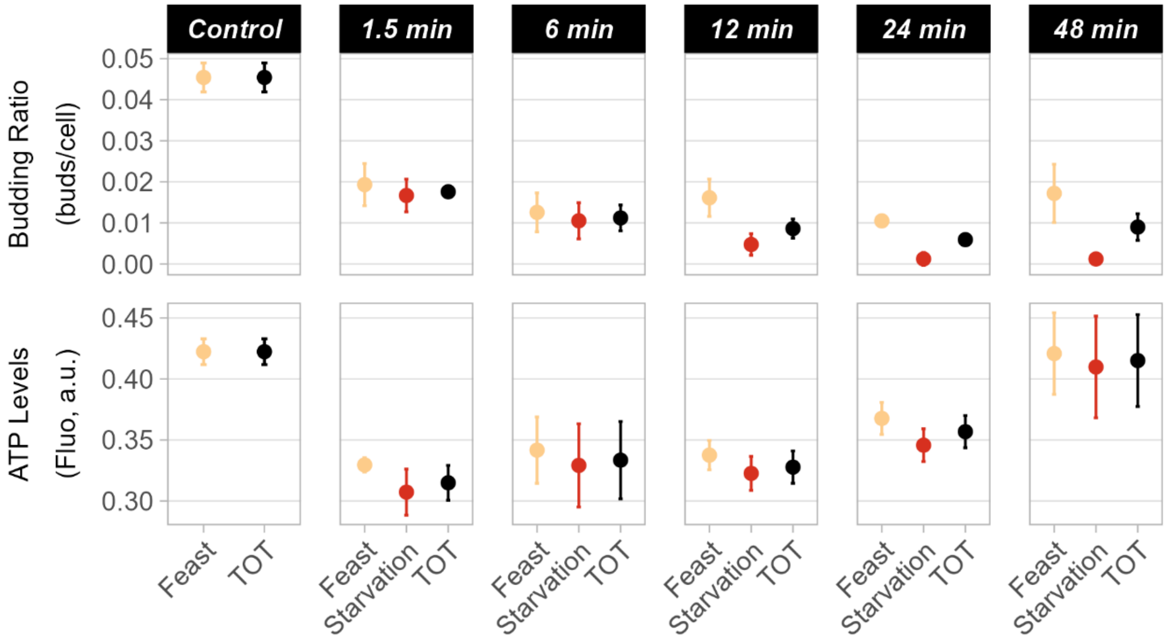

Supplementary Figure S6. Comparison of performance with respect to pulse. Performance data for budding ratio and ATP levels have been divided based on whether they were taken during a feast or starvation pulse. “TOT” refers to data for the whole screening. Dispersion of the data corresponds to the standard deviation across triplicates (three chambers).

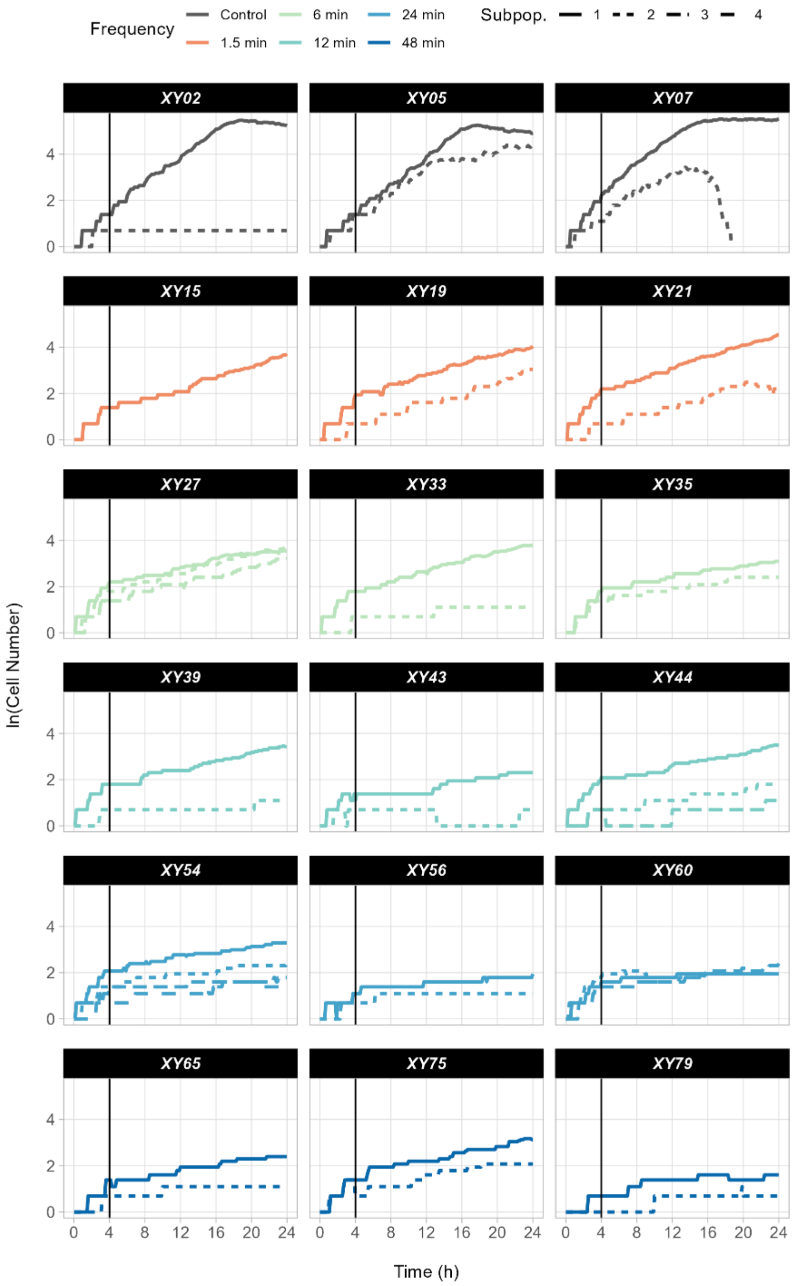

Supplementary Figure S7. Growth line plots for distinct cell subpopulations. For each replicate (i.e. chamber named "XY"), different subpopulations formed during the cultivation period are shown. Each line represents the subpopulation originating from an individual cell present at the beginning of the cultivation. Each chamber was inoculated with 1–4 cells.

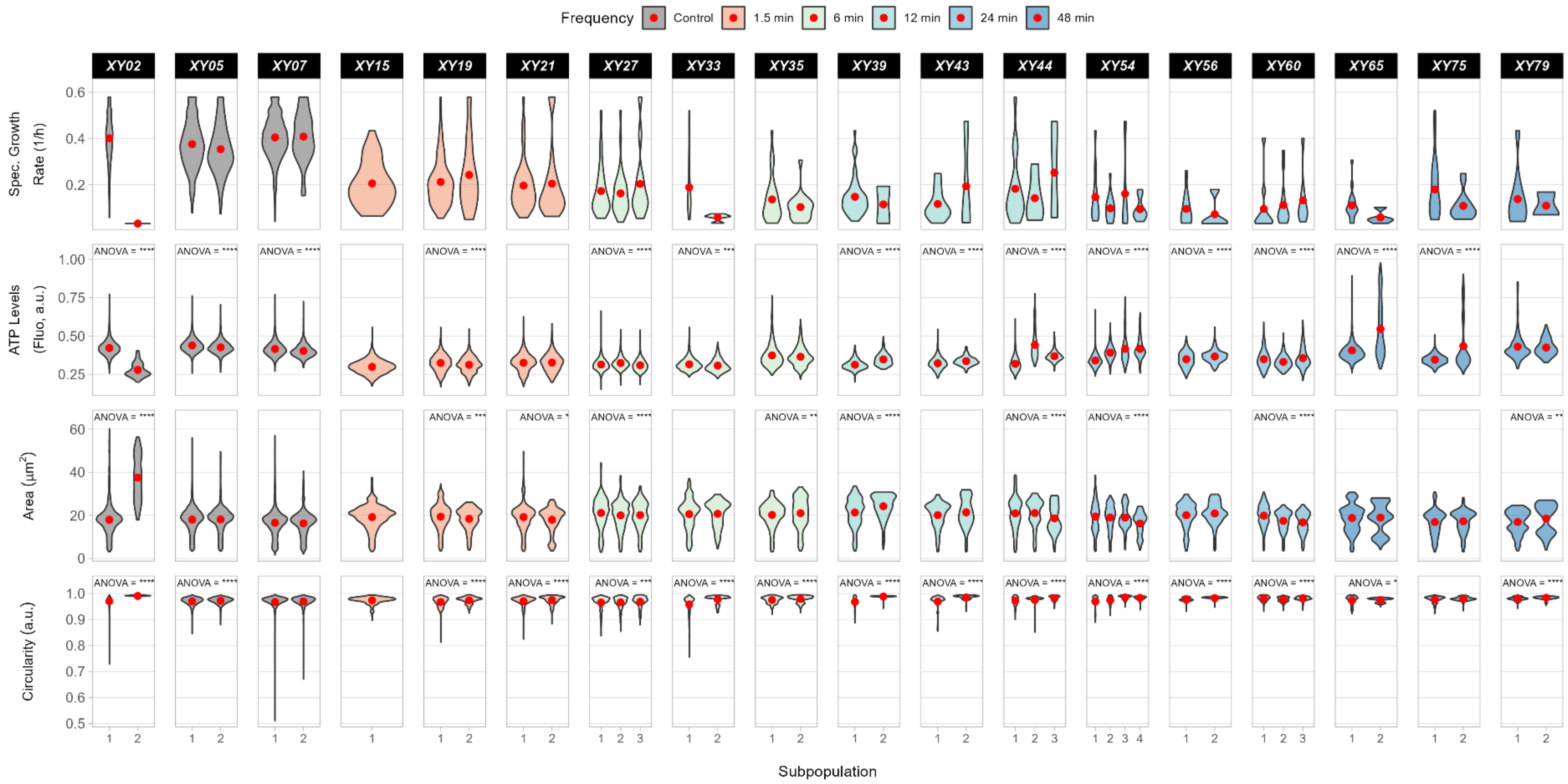

Supplementary Figure S8. Performance distribution of functions across subpopulation. For each chamber (named "XY"), subpopulations originating from an initial inoculum of 1–4 cells are shown. Violin plots present the single-cell performance for the following functions: specific growth rate, ATP levels, area, and circularity. The red dot in each violin plot represents the mean performance of that subpopulation. ANOVA was performed for each chamber to determine if the mean performances of subpopulations differed from one another; *p ≤ 0.05, **p ≤ 0.01, ***p ≤ 0.001, and ****p ≤ 0.0001.

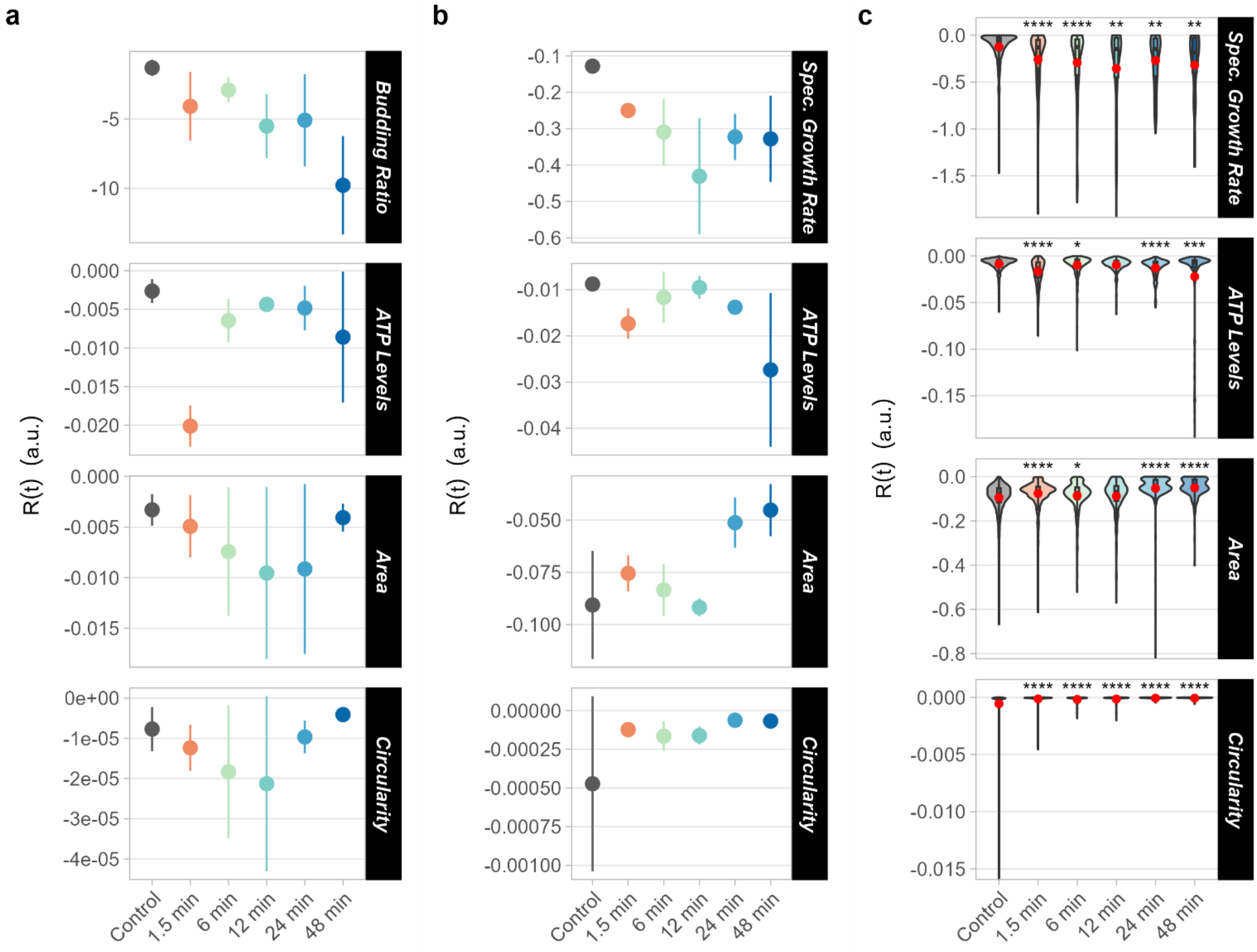

Supplementary Figure S9. Robustness over time. Robustness over time, R(t), denotes how stable a function is over time. Elevated R(t) values are associated with stable functions over time, while low R(t) values with unstable ones. R(t) was computed for the desired functions at the population (a) and single-cell (b) levels. The standard deviation refers to the distribution of triplicates (three chambers). (c) Distribution of R(t) for each individual cell. Red dots represent the mean R(t) for each condition. Student's *t*-test was used to evaluate the statistical difference of single-cell R(t) between each condition and the control; $^{*}p \leq 0.05$, $^{**}p \leq 0.01$, $^{***}p \leq 0.001$, and $^{****}p \leq 0.0001$.

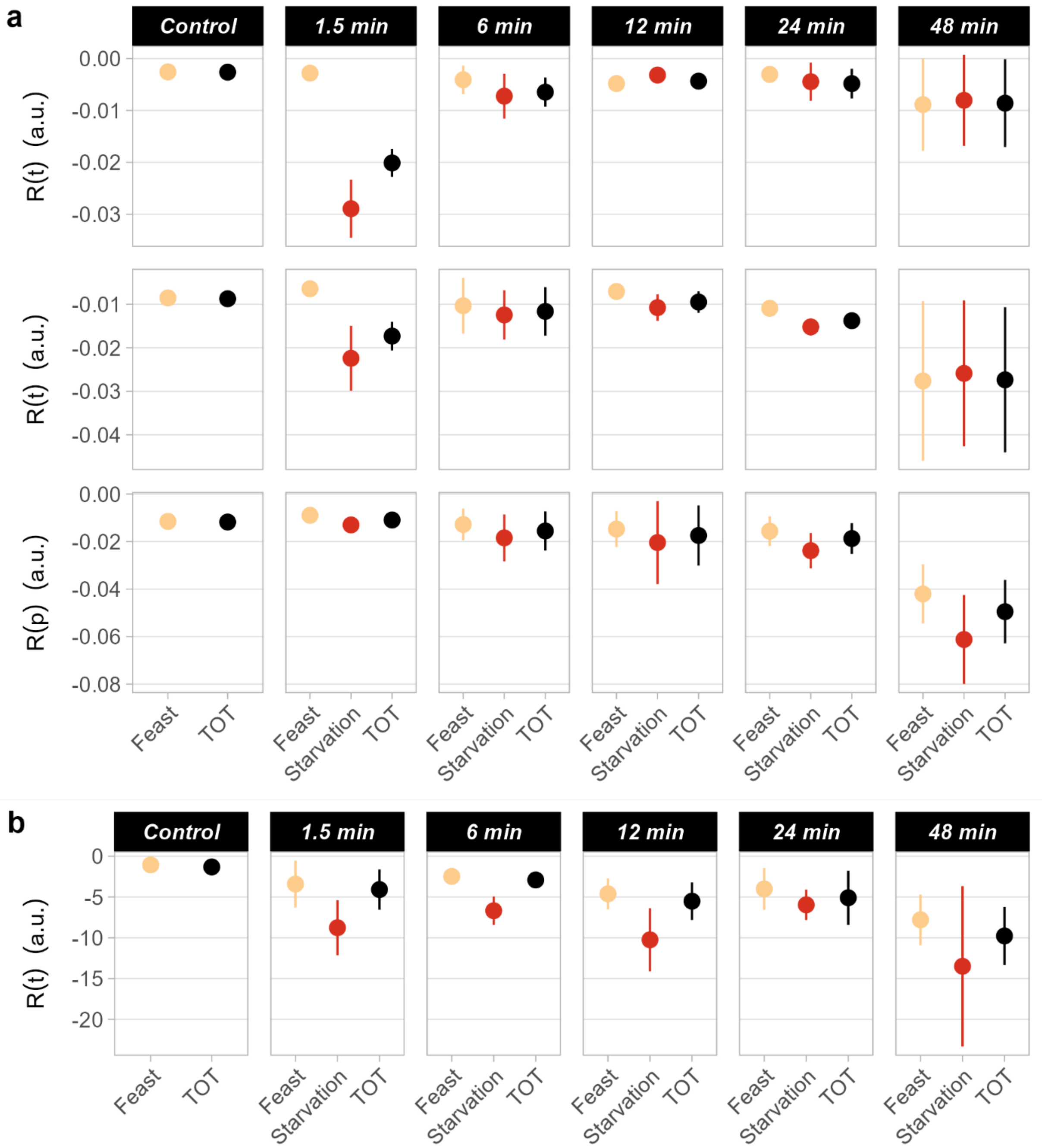

Supplementary Figure S10. Comparison of robustness with respect to pulse. Robustness data for ATP levels (a) and budding ratio (b) are categorised based on whether they were taken during a feast or starvation pulse. "TOT" refers to data for the whole screening. Dispersion of the data refers to the standard deviation across triplicates (three chambers). Robustness quantification was used to compute robustness over time, R(t), at population (panel a, top, and panel b) and single-cell levels (panel a, middle), as well as robustness across populations (panel a, bottom), R(p), to assess the stability of a function with respect to population heterogeneity.

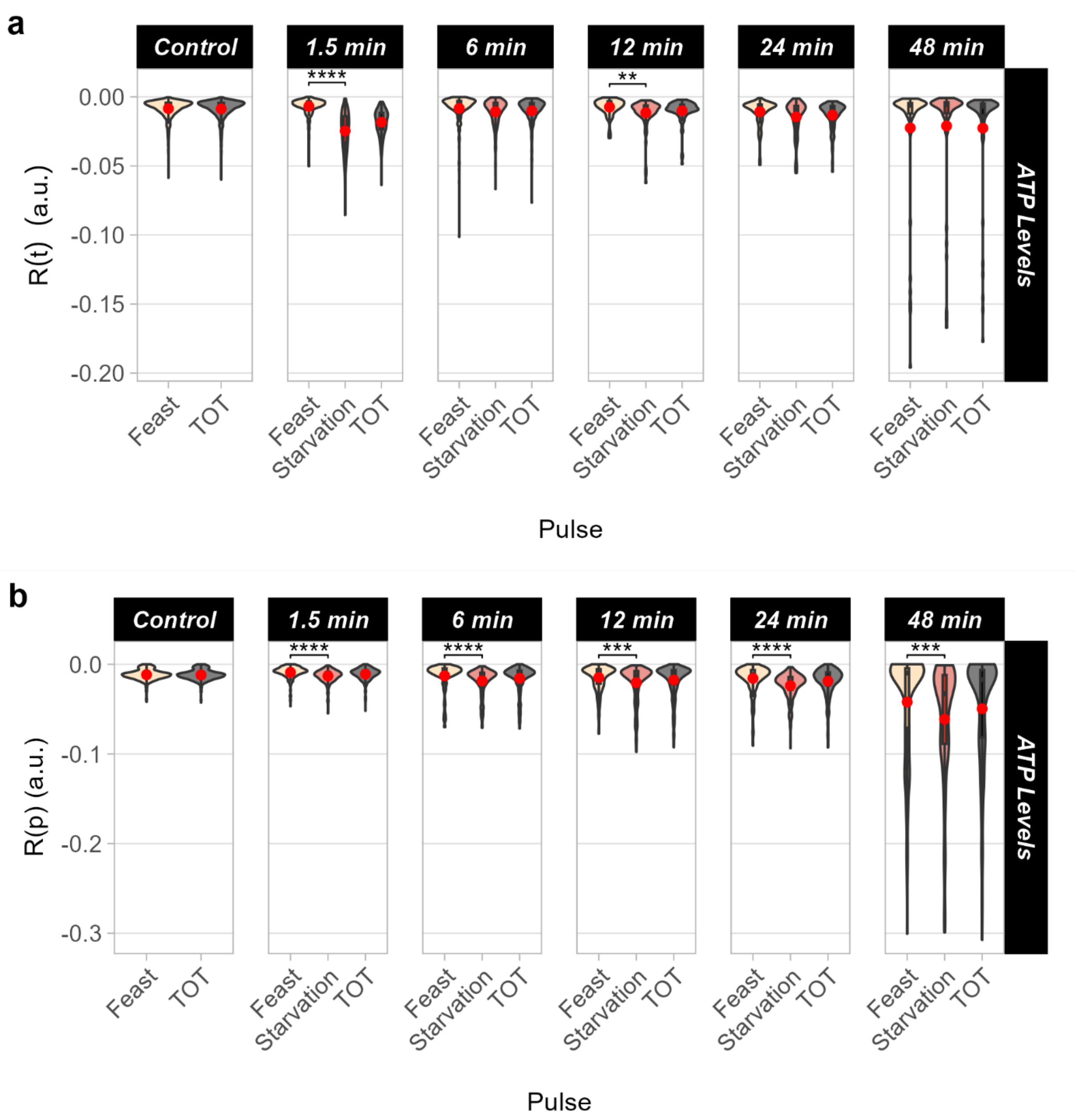

Supplementary Figure S11. Violin plots of robustness with respect to pulse. (a) Distribution of single-cell-level robustness over time for ATP content with respect to pulse (feast or starvation). "TOT" refers to data for the whole screening. Red dots represent the mean R(t) across all cells at each pulse. (b) Distribution of robustness across populations for ATP levels with respect to pulse (feast or starvation). "TOT" refers to data for the whole screening. Red dots represent the mean R(p) across all time points for each pulse. Student's *t*-test was used to evaluate the statistical difference of either R(t) or R(p) between starvation and feast pulses; *p ≤ 0.05, **p ≤ 0.01, ***p ≤ 0.001, and ****p ≤ 0.0001.

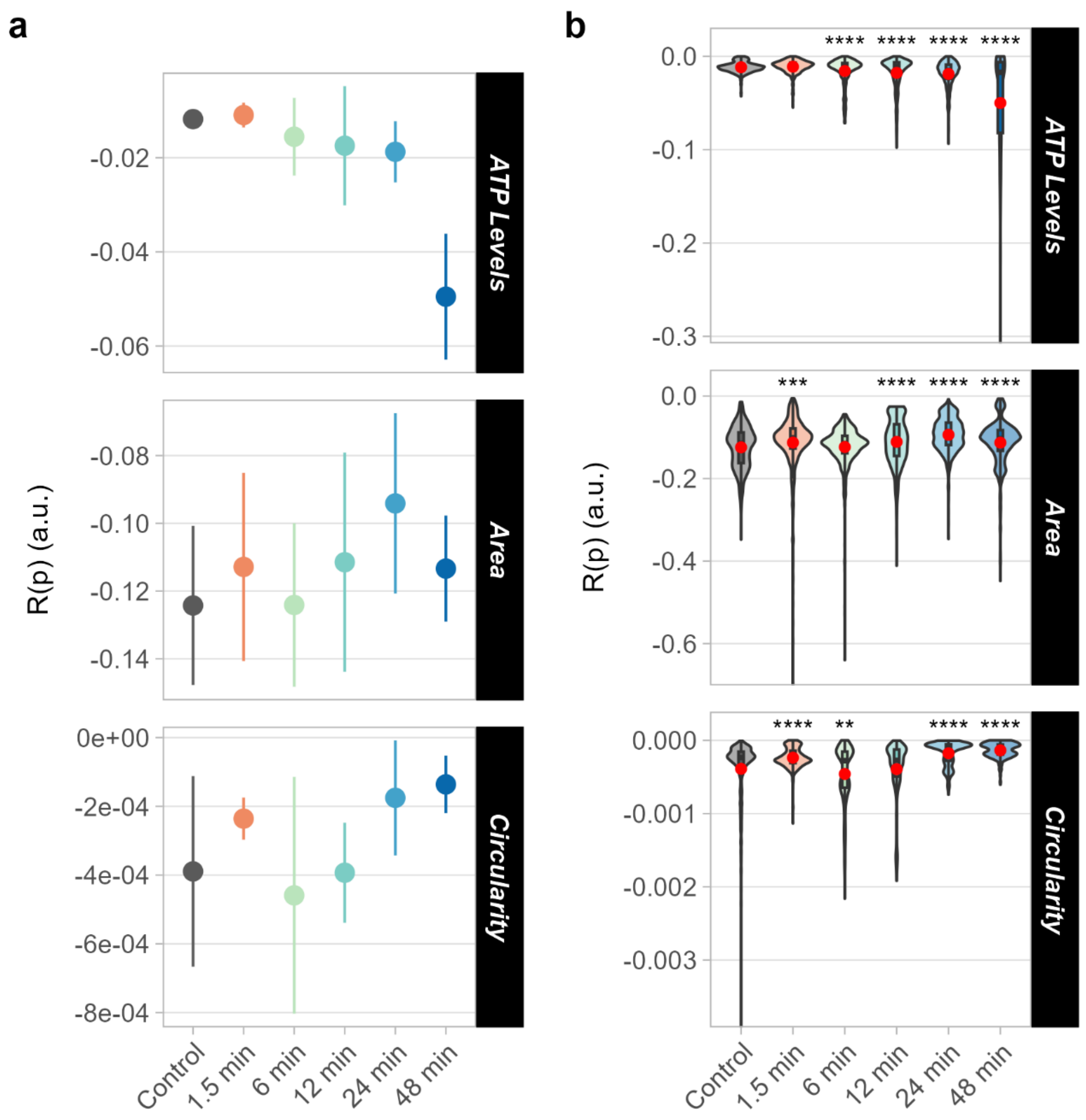

Supplementary Figure S12. Robustness across populations. Robustness across populations, R(p), denotes how stable a function is across a population at each time point. Elevated R(p) values are associated with homogeneous populations, while low R(p) values with heterogeneous ones. R(p) was computed for the desired functions (ATP levels, area, and circularity). (a) The standard deviation refers to the distribution across triplicates (three chambers). (b) Violin plots denote the distribution of R(p) for each time point. Each violin plot considers triplicates (three chambers) together. Data pertaining to each chamber are presented in Additional File 3. Red dots represent the mean R(p) across all time points for each condition. Student's *t*-test was used to evaluate the statistical difference between each condition and the control; *$p \leq 0.05$, **$p \leq 0.01$, ***$p \leq 0.001$, and ****$p \leq 0.0001$.

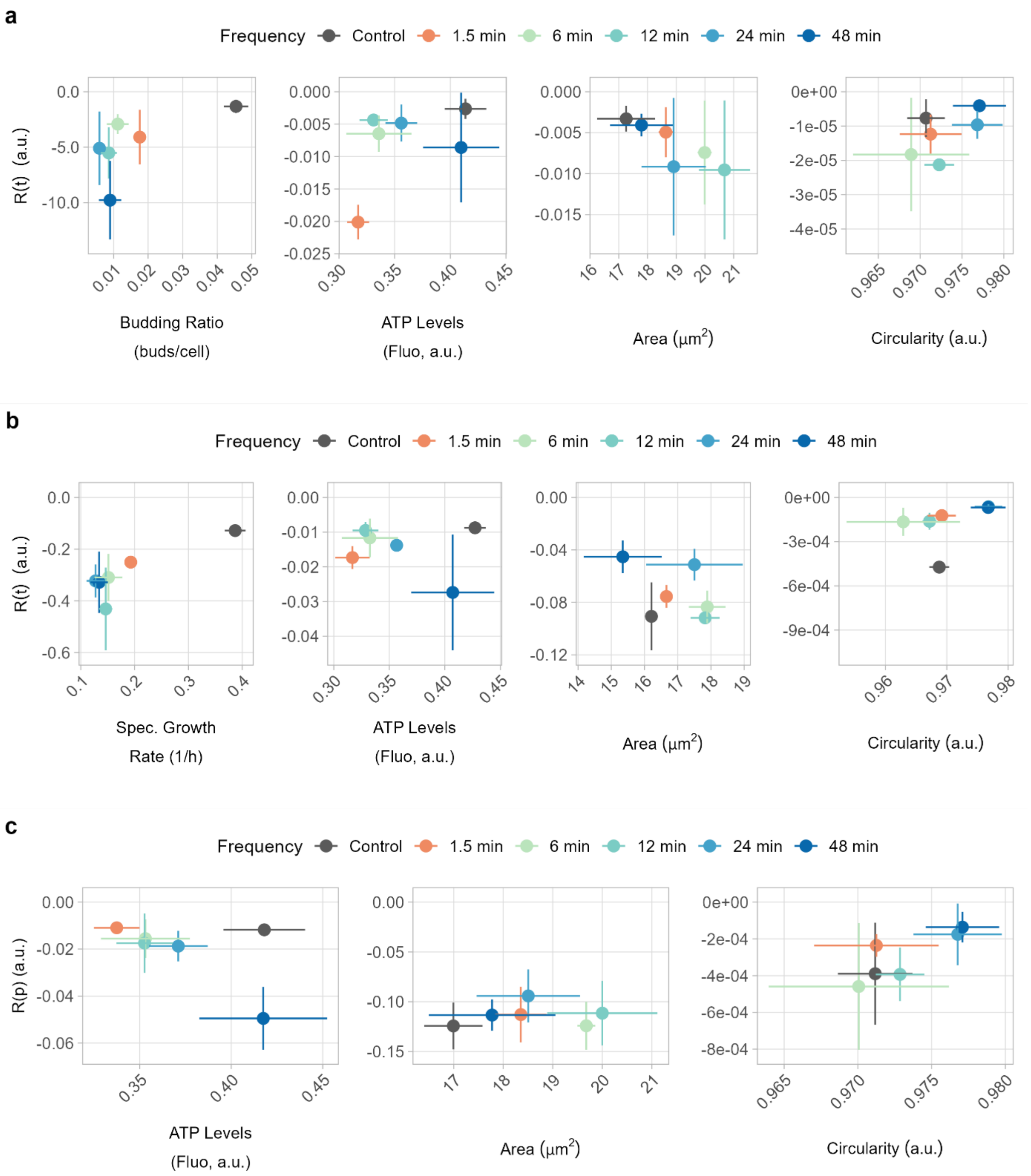

Supplementary Figure S13. Robustness vs performance plots. Correlation between performance and robustness over time at the population level (a) or robustness over time at the single-cell level (b), as well as robustness across populations (c) for selected cellular functions.
