# Supplementary material for: Quantifying microbial robustness in dynamic environments using microfluidic single-cell cultivation
Source: Microb Cell Fact. 2024 Feb 9;23:44. doi: 10.1186/s12934-024-02318-z (PMC10854032; doi:10.1186/s12934-024-02318-z)
Supplement: Supplementary file 3 — Additional file 3: Figure S1. Line plots for ATP levels over time. Line plots for ATP levels measured throughout the screening (24 h) in each chamber (named “XY”). Error bars refer to the standard deviation of the function at each time point across the entire cell population. The vertical line at 4 h identifies the beginning of the starvation-oscillation period. Figure S2. Line plots for growth curves. Line plots for growth curves measured throughout the screening (24 h) for each chamber (named “XY”). The vertical line at 4 h identifies the beginning of the starvation-oscillation period. Figure S3. Line plots for budding ratio over time. Line plots for the budding ratio measured throughout the screening (24 h) for each chamber (named “XY”). The vertical line at 4 h identifies the beginning of the starvation-oscillation period. Figure S4. Line plots for area over time. Line plots for the cellular area measured throughout the screening (24 h) for each chamber (named “XY”). Error bars refer to the standard deviation of the function at each time point across the whole cell population. The vertical line at 4 h identifies the beginning of the starvation-oscillation period. Figure S5. Line plots for circularity over time. Line plots for cellular circularity measured throughout the screening (24 h) for each chamber (named “XY”). Error bars refer to the standard deviation of the function at each time point across the whole cell population. The vertical line at 4 h identifies the beginning of the starvation-oscillation period. Figure S6. Violin plots showing the performance of individual chambers. Violin plots showing the distribution of performance data in individual chambers (named “XY”). “Merged” refers to the chamber triplicates considered altogether. The red dot denotes the mean across all cells in that chamber. The dashed horizontal line is the mean of the merged chambers. Figure S7. Violin plots showing robustness over time of individual chambers. Violin plots showing [file 12934_2024_2318_MOESM3_ESM.pdf]

Additional File 3

Quantifying microbial robustness in dynamic environments using microfluidic single-cell cultivation

Luisa Blöbaum[§1,2], Luca Torello Pianale[§3], Lisbeth Olsson[3*], Alexander Grünberger[*1,4]

[1]Multiscale Bioengineering, Technical Faculty, Bielefeld University, Bielefeld, Germany

[2]CeBiTec, Bielefeld University, Bielefeld, Germany

[3]Industrial Biotechnology Division, Department of Life Sciences, Chalmers University of Technology, Gothenburg, Sweden

[4]Microsystems in Bioprocess Engineering, Institute of Process Engineering in Life Sciences, Karlsruhe Institute of Technology, Karlsruhe, Germany

[§]Equal contribution

*Correspondence: Alexander Grünberger (alexander.gruenberger@kit.edu).

Supplementary Information

The “Supplementary Figures” section includes:

- Line plots for ATP levels over time (Supplementary Figure S1).
- Line plots for growth curves (Supplementary Figure S2).
- Line plots for budding ratio over time (Supplementary Figure S3).
- Line plots for area over time (Supplementary Figure S4).
- Line plots for circularity over time (Supplementary Figure S5).
- Violin plots for the performance of individual chambers (Supplementary Figure S6).
- Violin plots for robustness over time of individual chambers (Supplementary Figure S7).
- Violin plots for robustness across populations of individual chambers (Supplementary Figure S8).

Supplementary Figures

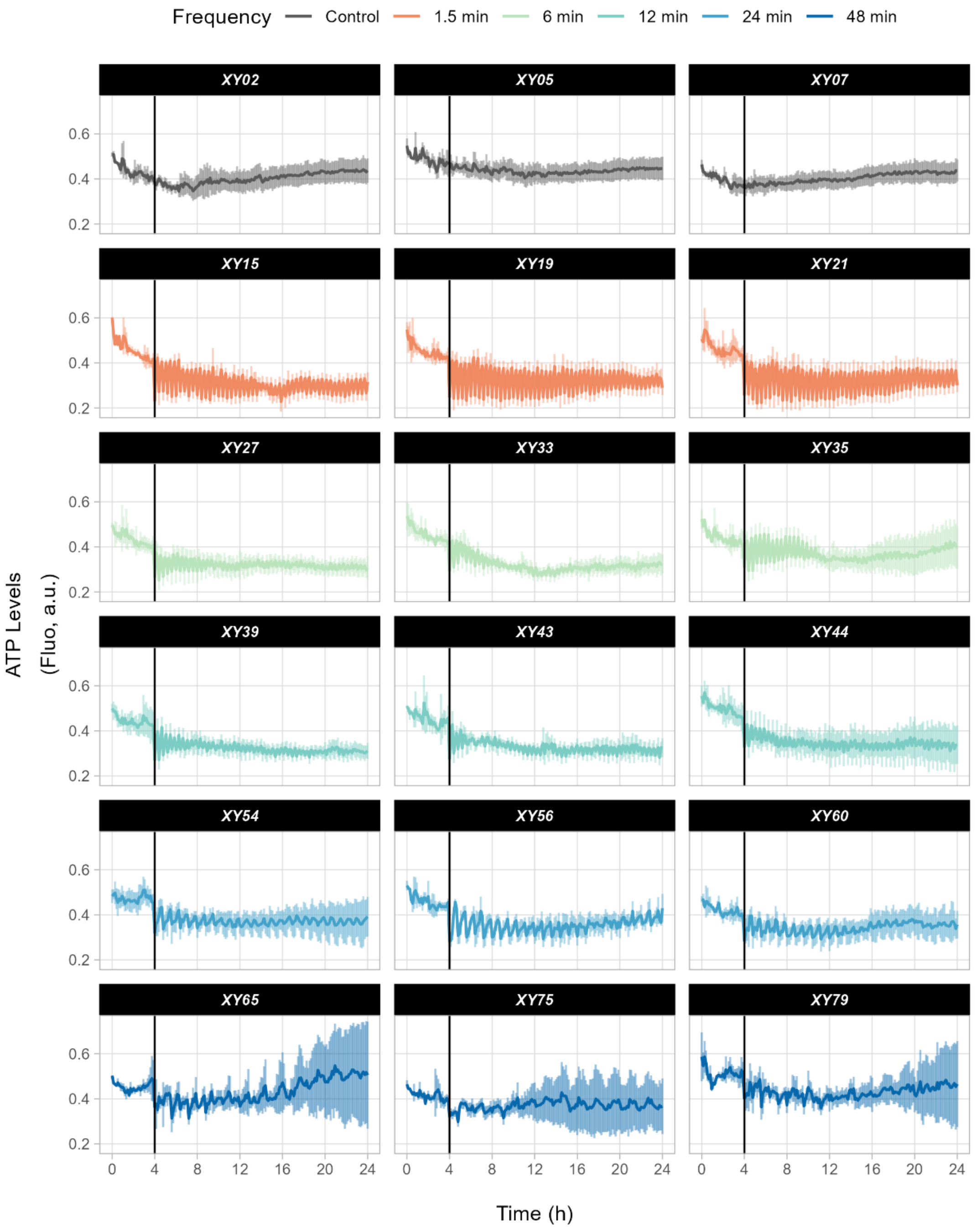

Supplementary Figure S1. Line plots for ATP levels over time. Line plots for ATP levels measured throughout the screening (24 h) in each chamber (named “XY”). Error bars refer to the standard deviation of the function at each time point across the entire cell population. The vertical line at 4 h identifies the beginning of the starvation-oscillation period.

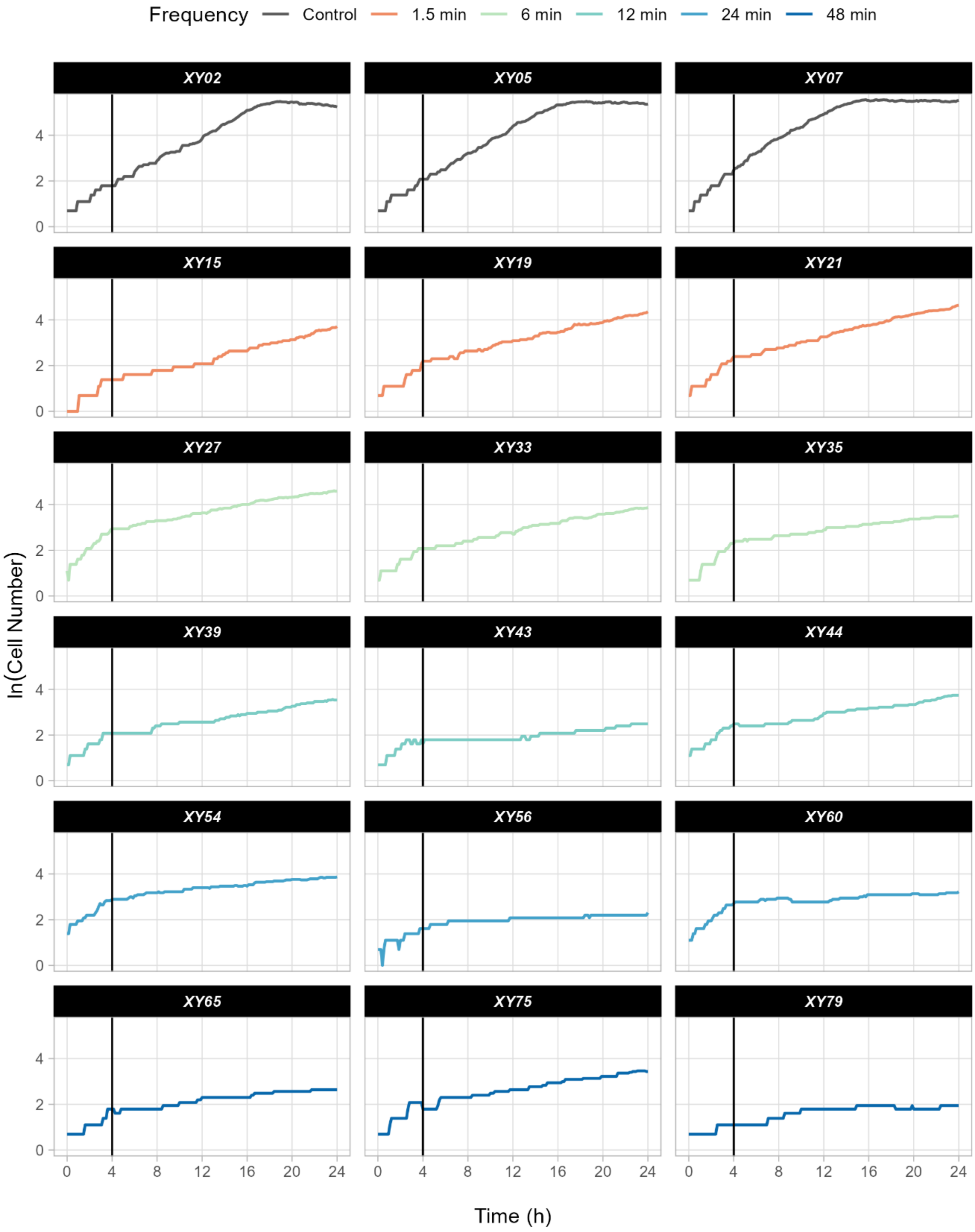

Supplementary Figure S2. Line plots for growth curves. Line plots for growth curves measured throughout the screening (24 h) for each chamber (named "XY"). The vertical line at 4 h identifies the beginning of the starvation-oscillation period.

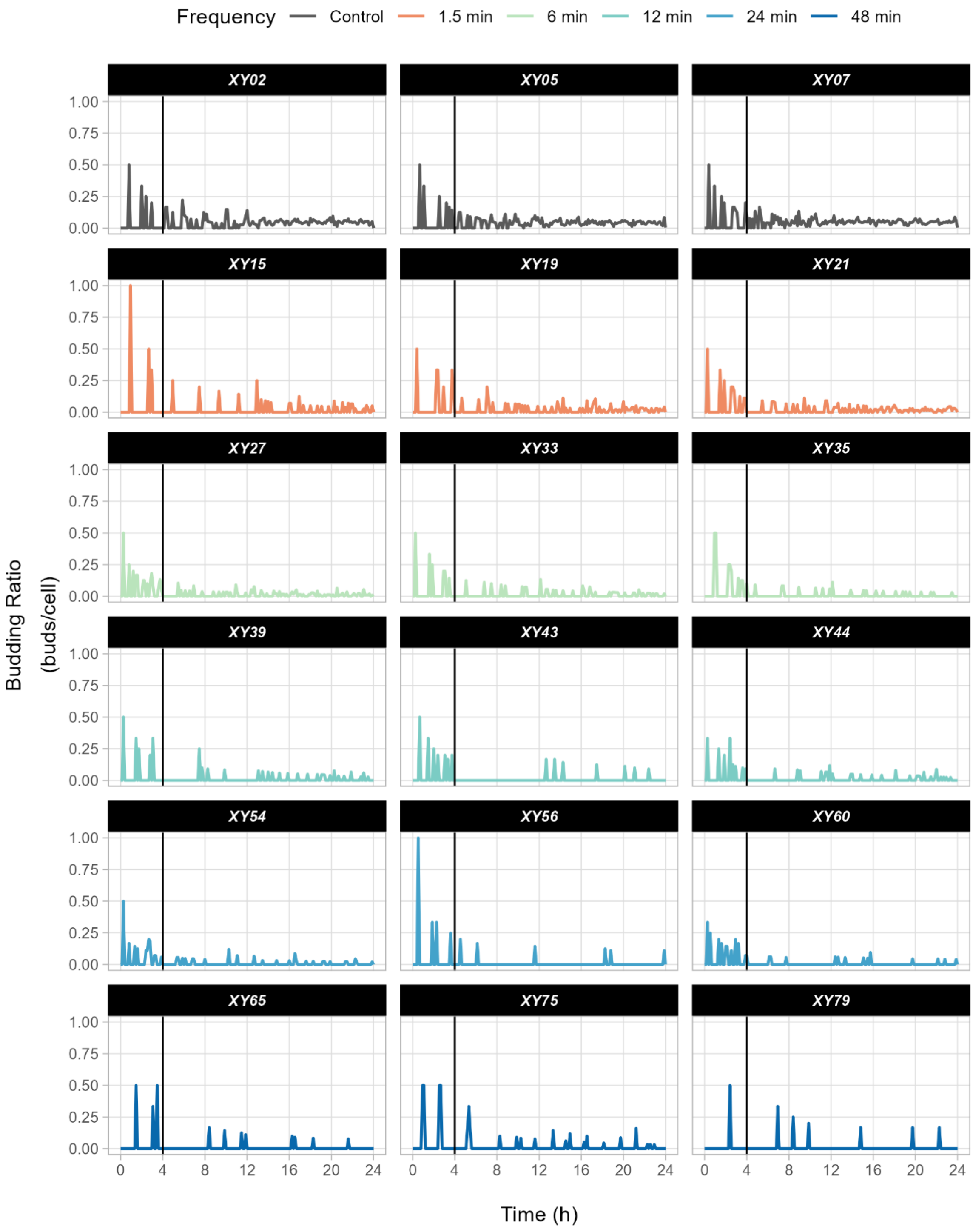

Supplementary Figure S3. Line plots for budding ratio over time. Line plots for the budding ratio measured throughout the screening (24 h) for each chamber (named "XY"). The vertical line at 4 h identifies the beginning of the starvation-oscillation period.

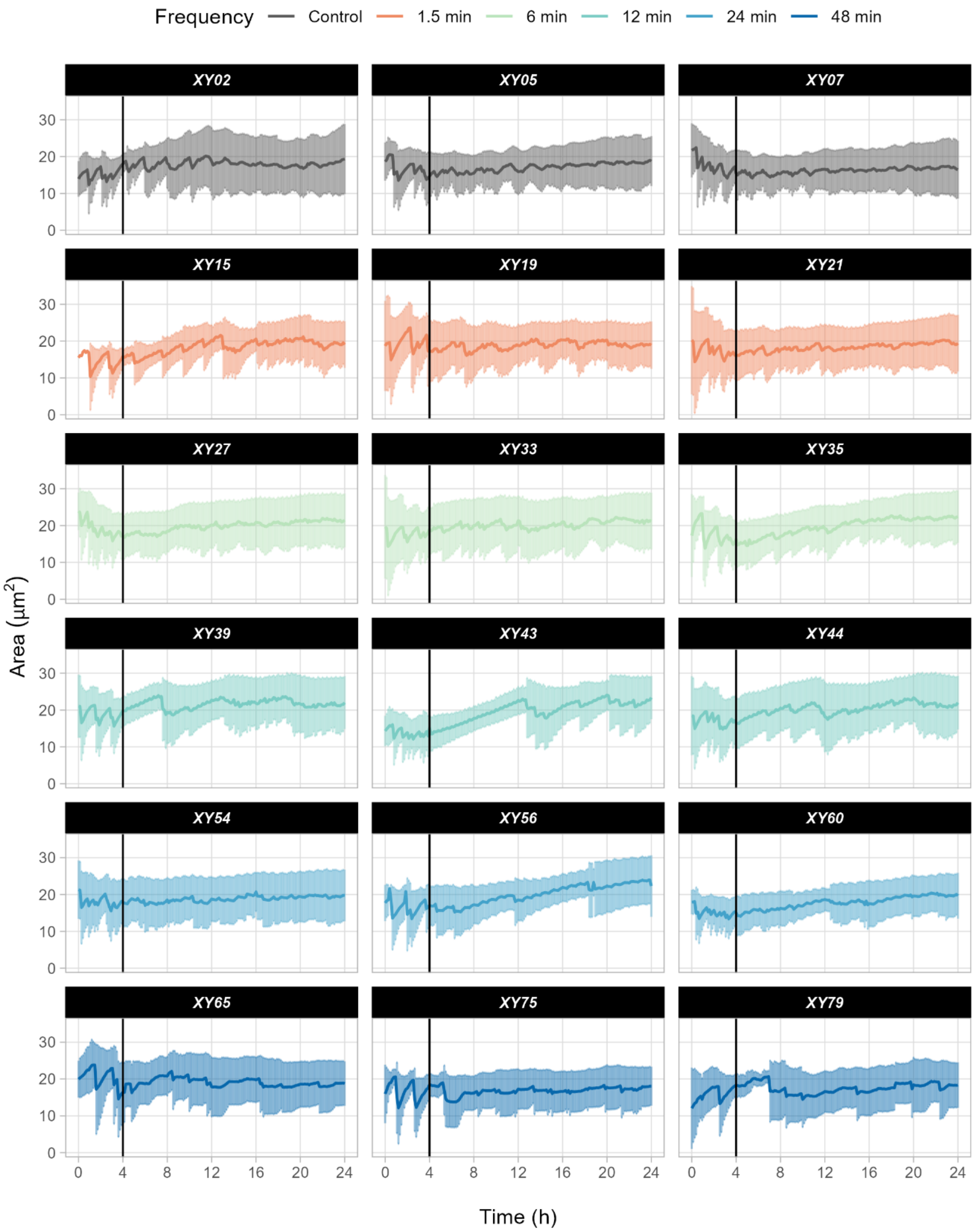

Supplementary Figure S4. Line plots for area over time. Line plots for the cellular area measured throughout the screening (24 h) for each chamber (named "XY"). Error bars refer to the standard deviation of the function at each time point across the whole cell population. The vertical line at 4 h identifies the beginning of the starvation-oscillation period.

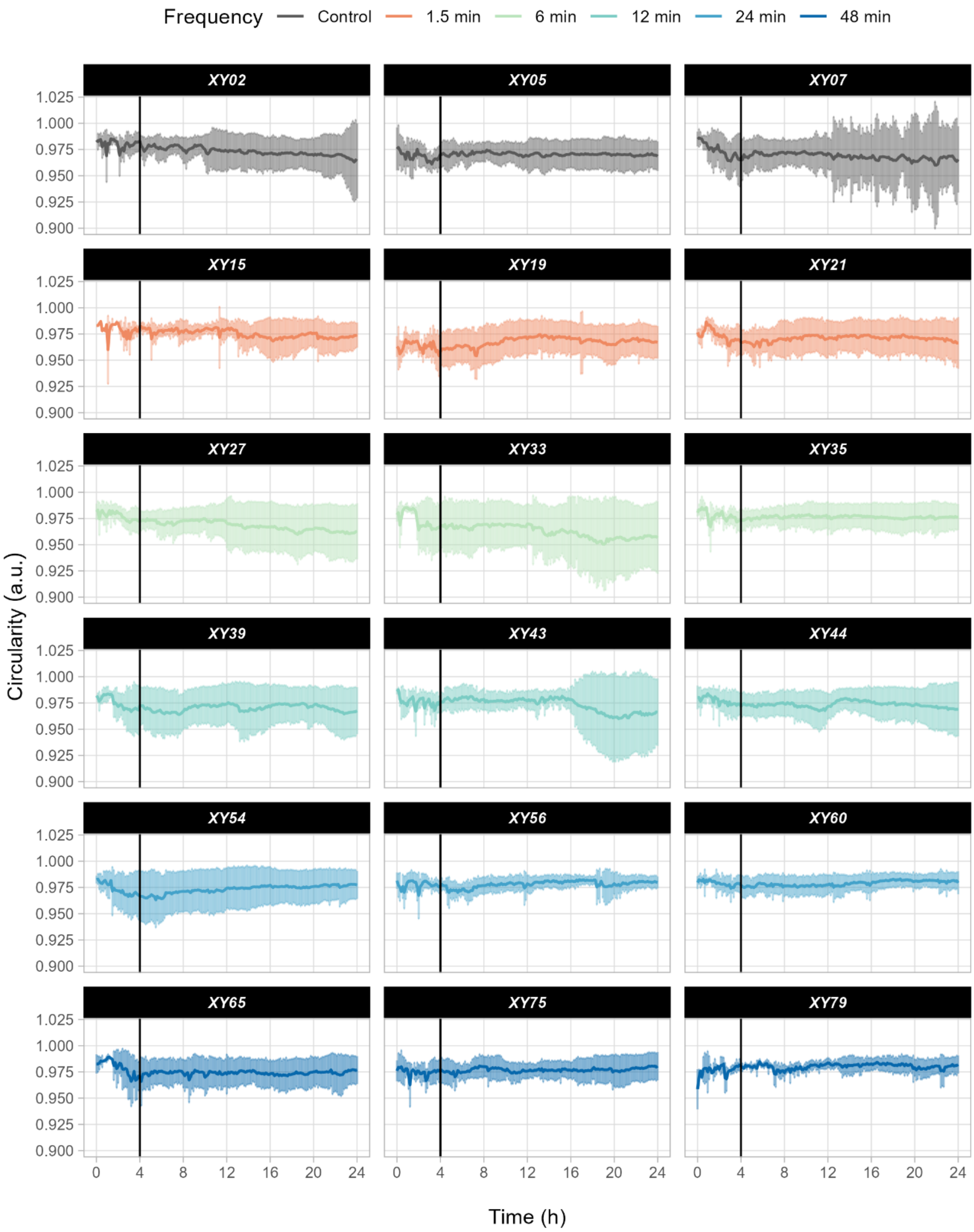

Supplementary Figure S5. Line plots for circularity over time. Line plots for cellular circularity measured throughout the screening (24 h) for each chamber (named "XY"). Error bars refer to the standard deviation of the function at each time point across the whole cell population. The vertical line at 4 h identifies the beginning of the starvation-oscillation period.

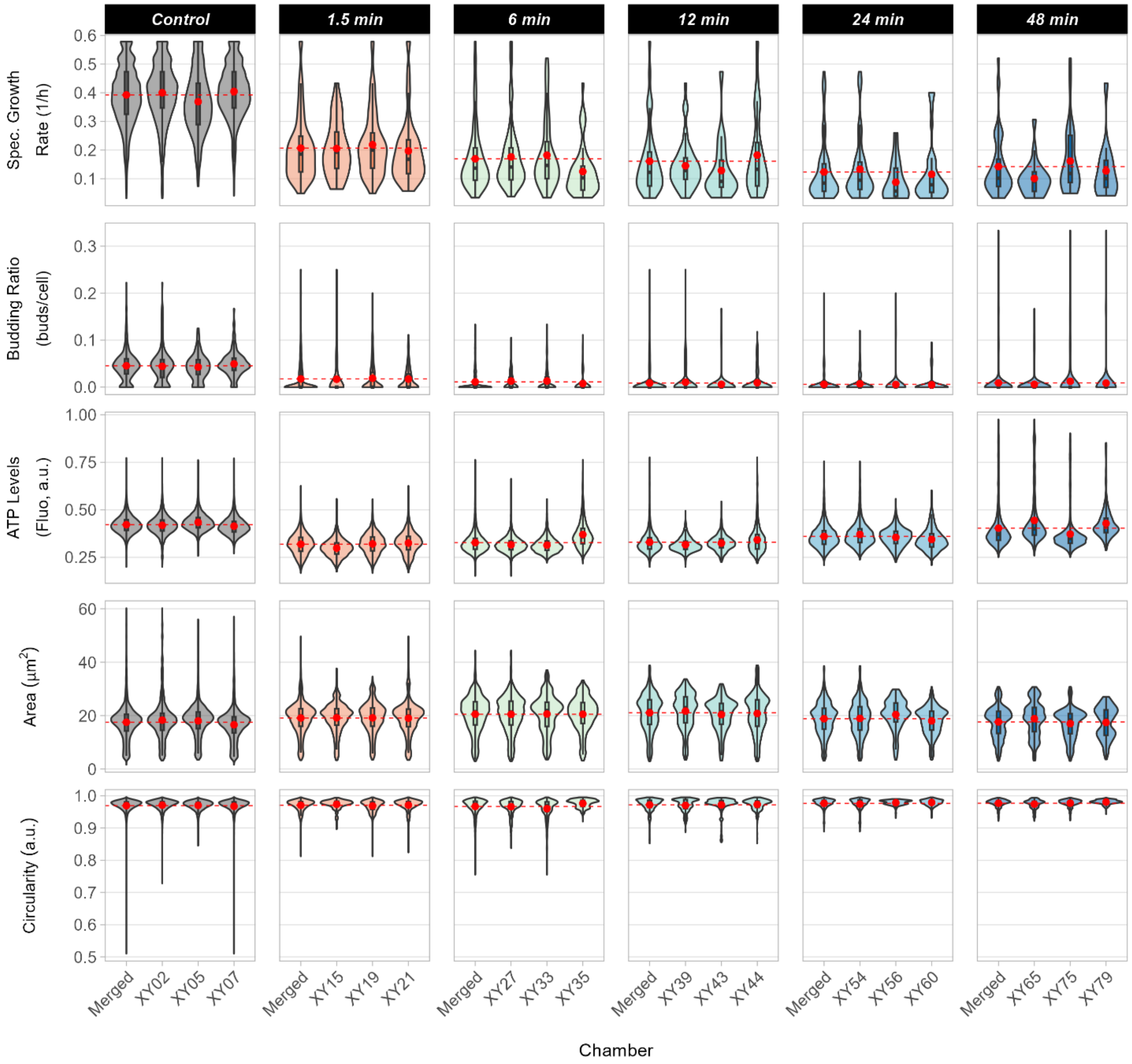

Supplementary Figure S6. Violin plots showing the performance of individual chambers. Violin plots showing the distribution of performance data in individual chambers (named "XY"). "Merged" refers to the chamber triplicates considered altogether. The red dot denotes the mean across all cells in that chamber. The dashed horizontal line is the mean of the merged chambers.

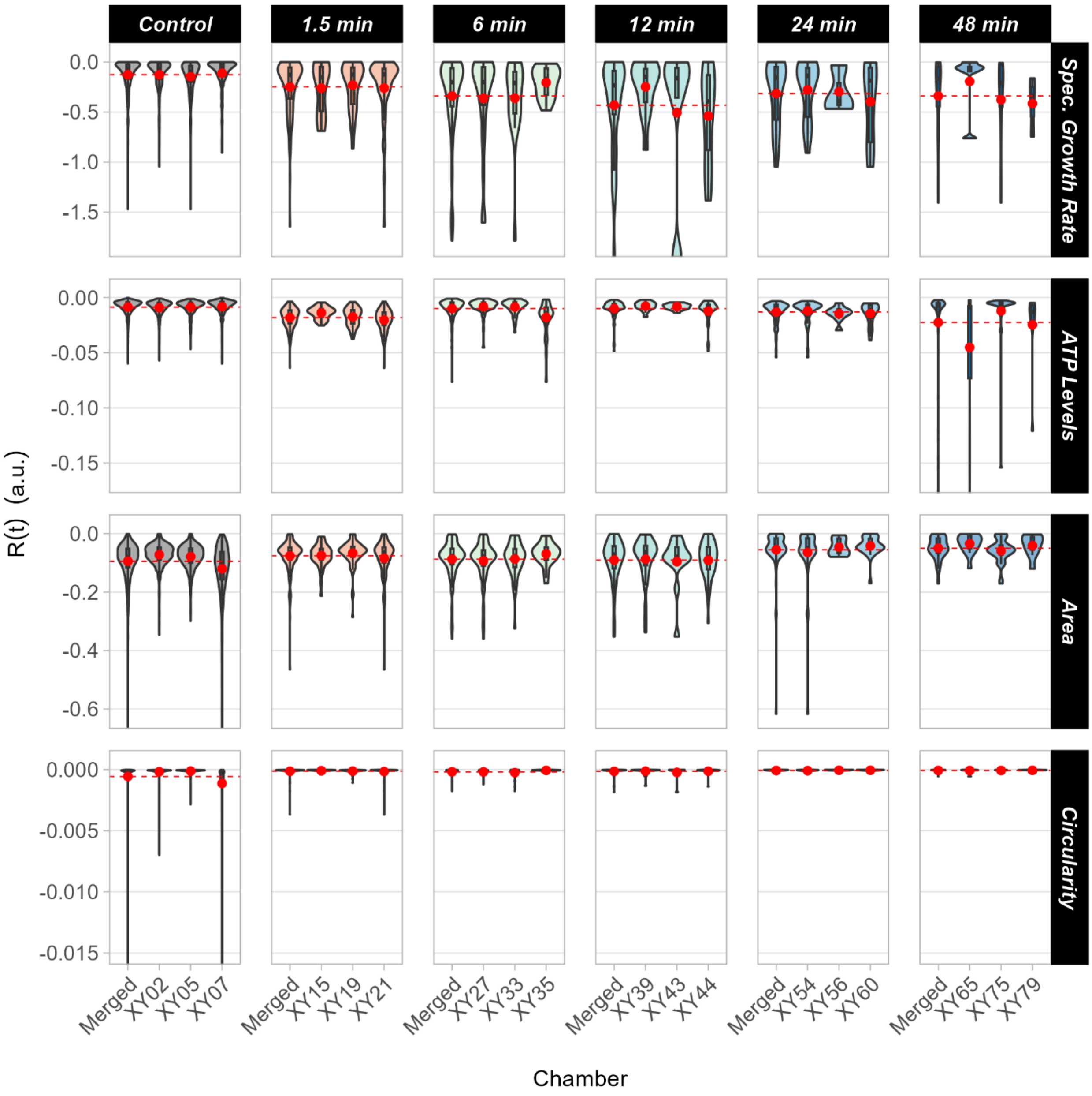

Supplementary Figure S7. Violin plots showing robustness over time of individual chambers. Violin plots showing the distribution of data for robustness over time at single-cell level in individual chambers (named “XY”). “Merged” refers to the chamber triplicates considered altogether. The red dot denotes the mean across all cells in that chamber. The dashed horizontal line is the mean of the merged chambers.

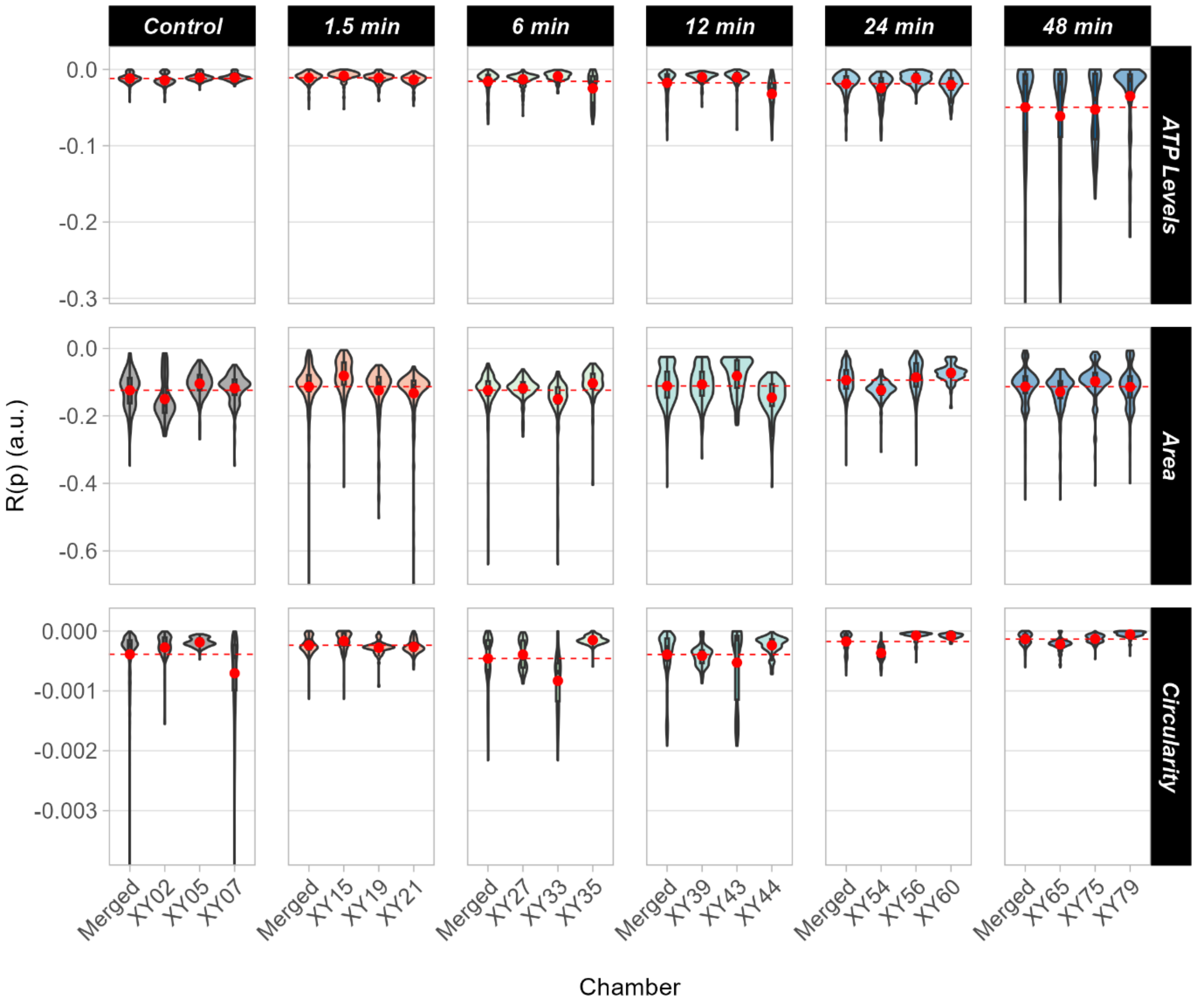

Supplementary Figure S8. Violin plots showing robustness across populations in individual chambers. Violin plots showing the distribution of data for robustness across populations in individual chambers (named "XY"). "Merged" refers to the chamber triplicates considered altogether. The red dot denotes the mean across all cells in that chamber. The dashed horizontal line is the mean of the merged chambers.
